# Supplementary material for: The pocketome of G-protein-coupled receptors reveals previously untargeted allosteric sites
Source: Nat Commun. 2022 May 10;13:2567. doi: 10.1038/s41467-022-29609-6 (PMC9091257; doi:10.1038/s41467-022-29609-6)
Supplement: Supplementary file 1 — Supplementary Information [file 41467_2022_29609_MOESM1_ESM.pdf]

The pocketome of G-protein-coupled receptors reveals previously untargeted allosteric sites
Supplementary Information

Janik B. Hedderich[1], Margherita Persechino[1], Katharina Becker[2], Franziska M. Heydenreich[3,4], Torben Gutermuth[1], Michel Bouvier[4], Moritz Bünemann[2], Peter Kolb[1,*]

[1]Department of Pharmaceutical Chemistry, Philipps-University Marburg, Marburg, Germany
[2]Department of Pharmacology & Clinical Pharmacy, Philipps-University Marburg, Marburg, Germany
[3]Department of Molecular and Cellular Physiology, Stanford University School of Medicine, Stanford, CA, USA
[4]Institute for Research in Immunology and Cancer, Department of Biochemistry and Molecular Medicine, Université de Montréal, Montréal, QC, Canada
[*]to whom correspondence should be addressed: peter.kolb@uni-marburg.de

Supplementary Notes

Probe Docking

On average, 2 500-3 300 poses were obtained for each of the receptor structures we docked to. While the lowest number of poses was obtained for PDB 2J4Y (1 776), the highest number was obtained for PDB 4IAR (3 808). This indicates a number of probe poses for all receptors sufficient for further analysis. In a probe-based view, benzimidazole showed the highest average number of poses per structure, while favourable poses for the trimethyl ammonium cation were extracted least frequently (Supplementary Table 1 and Supplementary Figure 1). No poses were found for methanamine in PDB 4PY0 and the tetramethyl ammonium cation in PDB 4OO9. At the other end, the highest number of poses (200) was obtained for benzimidazole in PDB 6FUF, β-lactam in PDB 6UP7 and indoline in PDB 6FUF. All receptor structures were able to host both apolar and polar probes, with no clear trends discernible.

General Distribution of Pockets

Starting on the outwards-facing portion of the receptor in the region between helices I and II, we identified two regions of interest on the upper and lower portion of the 7TM bundle, referred to as OS1 and OS2, respectively. While OS2 only shows small and scattered densities for classes A, B1, B2, C and D1, OS1 is more defined for classes A and B1. The density maps of classes B2, C, D1 and F show smaller volumes for OS1.

Next, between helices II, III and IV, a well-defined, large density in front of the extracellular ends of helices II and III is visible for classes A, B1, C and F (KS1), while the map only shows a small hotspot for classes B2 and D1. Adjacent to KS1, we identified another site in front of helices III and IV for classes A, B1, B2, C and D1, referred to as KS2. The class F map only shows a smaller, scattered density in this region. Below KS2, the third site

of this region, KS3, resides between helices II and IV and appears to be conserved in all of the analysed GPCR classes. For classes A, B1, C and D, KS3 even reaches across helix II down to the intracellular end of helix I near OS2. Notably, the densities of KS2 and KS3 are merged for classes A and B1, while class C, D1 and F show well-separated spots.

The extracellular region at the lateral portion of the 7TM bundle between helices III, IV and V again includes three separate densities. At the upper end, KS4 resides between helices IV and V for classes A, B1, B2, D1 and F. Class C only shows a smaller density that is shifted towards helix V. Below, class A features a large hotspot leaning against helices III and IV (KS5). In direct comparison, the class B1 density in this region is similar in size, but split up into two sub-sites. For classes B2, C, and D1 the densities of KS5 are smaller. The third site, KS6, is located adjacent to KS5 only for classes A, B1 and C. In the density map for class B1, these two sites are connected. The density maps for classes D1 and F show a site that is shifted towards helix V.

Lastly, we identified a larger region of interest in front of helices I, VI, VII and VIII. For class B1, these densities are connected across helix V with the sites described before. Two densities reside in the upper portion of the 7TM bundle, one between helices VI and VII and one in front of helix VII (OS6 and OS7). These two sites seem to be conserved across almost all classes except B2 and D1, where only OS7 is visible. For classes A, B1 and C, the densities of OS7 are shifted towards helix I. Right below OS6 and OS7, two other sites reside at the middle portion of helices VI and VII, referred to as KS7 and OS8. Again, these two sites are conserved across all analysed classes besides class D1. In general, the densities of KS7 and OS8 are rather small and spotty. Here, one exception is OS8 of class F, which shows a large and well-defined hotspot. The last sites described for the outwards-facing receptor regions are located in the lower portion of the 7TM bundle. The first site, KS8, resides between helices VI and VII below KS7. While the maps for classes A, B1, D1 and F show well-defined spots, the densities of class C are rather scattered. For class B2, we could not identify any density near this region. Notably, the densities of KS7 and KS8 are merged for class B1. The next site, KS9, is located around the kink between helices VII and VIII. Regarding its size and position, this spot differs a lot between the classes. While classes C and D1 show larger spots in this region, the densities of classes A and B1 are quite small. The maps of classes B2 and F, on the other hand, do not show any density in this region. Finally, the last spot, OS10, is located next to OS9 between helices I and VIII. Here, a well-defined density is observable for all classes except B2 and D1.

In the vicinity of the orthosteric pocket, two secondary binding pockets are observable. The first of these secondary pockets described here, IBS2, shows a lengthy density reaching into the extracellular loops near helices IV, V and VI. While this spot seems to be very specific for class A, some scattered density can also be seen for class C. Class B2 also shows a smaller spot above its orthosteric site. Next, IBS3 is located adjacent to the orthosteric site between helices I, II, VI and VII. This region clearly is conserved in classes A and B1, and also the class C density indicates the existence of a secondary site. Interestingly, the location of the orthosteric site and IBS3 seem to be switched for class D1. Going down towards the intracellular side, a small density, KS10, is visible below the orthosteric site for class B1. Notably, the class B2 map shows a large spot in this region.

Identification of Known Pockets

A similar picture as for KS2 emerges for KS5 and KS8. The former resides at the outwards-facing portion of the receptor between helices III, IV and V and is known from class A structures of the FFAR1, C5AR1 and β_2AR. While the approximate location of this site is conserved across all GPCR classes in the density maps, the shapes differ substantially. This is consistent with the similarity matrix, which reveals an overall lower sequence similarity among the different classes (Supplementary Figure 5). In direct comparison, the physico-chemical properties of KS8 tend to be conserved for classes A and B1. While some class C GPCRs share a high sequence homology to classes A and B1, the properties of class F KS8 are dissimilar to those of the other classes. The densities, however, show up at the lower portion of the 7TM bundle in front of helices VI and VII in all classes. So far, KS8 is known only from class B1 and class C structures.

In the case of KS5, we focused on β_2AR structures, one of them resolved with a PAM (PDB 6N48), the other structure including a NAM (PDB 6OBA) in this region. As seen in Supplementary Figure 8, KS5 is mainly apolar with the exception of a few residues. Both allosteric modulators described in their respective structures fill different portions of this binding site: While the NAM is located at the upper part of KS5, the PAM resides at the lower end of the 7TM bundle. By looking at the contact analysis of KS5 (Supplementary Figure 7), we found that the residue contacts of helices III, IV and V are mainly important for an active conformation of the receptor. Hence, the region around KS5 seems to cluster together upon activation.

By the time of this analysis, only NAMs are known to modulate the class B1 receptors GLP1R and GLR by addressing KS8, namely ligands PF-06372222, NNC0640 and MK-0893. On the other hand, one PAM binding to this site is known for the class C receptor GABR2 (ligand BHFF). The comparison of inactive and active state class B1 structures shows that the NAMs seem to interfere with the outward movement of helix VI away from helix VII. Our residue contact analysis for B1 structures (Supplementary Figure 14) revealed multiple contacts crucial for both an active and inactive state of the receptor.

Identification of Orphan Pockets

For OS6, the sequence similarity matrix (Supplementary Figure 5) shows another interesting pattern. In this region, class A receptors share sequence homology with classes C and F to some extent. Class C and F GPCRs by themselves, however, differ from each other in this region. Regarding OS6, class B1 receptors only show sequence homology within their class. The densities located at the upper portion of the GPCR between helices VI and VII were conserved across all classes besides B2 and D1. Our contact analysis for class A and B1 orphan sites shown in Supplementary Figures 10 and 15 reveals multiple contacts conserved for both an active or inactive conformation of the receptor.

Supplementary Discussion

In order to describe a plausible modulation through KS5, we focused on two β_2AR structures. The particular difference in the binding mode of the allosteric modulators could give insight

into the modulation of GPCRs through KS5. By looking at the contact analysis of KS5 (Supplementary Figure 7), we found that the residues of helices III, IV and V cluster together upon activation. Hence, addressing the lower portion of KS5 with a ligand acting as a hydrophobic patch could stabilise the formation of active state contacts. This mode of action would be comparable to the stabilisation of an inactive state through KS2. The modulation towards an inactive state through KS5, on the other hand, could be achieved by addressing residue 3.41 in particular. Our contact analysis reveals that besides its major involvement in active state contacts, this residue forms a highly conserved contact in inactive state structures (3.41-4.53). As seen in the inactive β_2AR structure, the NAM interacts with 3.41. In direct comparison to the active structure, this interaction seems to keep the contact between 3.41 and 4.53 intact, which might prevent the formation of the active residue clustering downstream.

In order to elucidate possible modes of action for KS8 in class B1 receptors in more detail, we analysed the interaction patterns of three structures (PDBs 5VEW, 5VEX, 5EE7) and compared them to our class B1 contact analysis (Supplementary Figure 14). We found that all of the NAMs inserted themselves between the lower regions of helices VI, VII, and VIII. Our B1 contact analysis reveals that a ligand might disrupt important active state contacts that way. Since no complex structure of a PAM with a class B1 GPCR is known, we can only hypothesise about the possible positive allosteric modulation of these receptors. A possible design concept is to address the upper part of the site and disrupt crucial inactive state contacts. Also, analysing the binding mode of the class C PAM does not give further insights into the positive modulation of GPCRs through KS8. This is plausible, since this ligand seems to act as a stabiliser for the functionally important dimerisation of the GABR2 instead of directly interfering with active or inactive residue contacts.

The shapes and sequence similarities of OS6 across all analysed GPCR classes showed patterns that might be interesting for the design of selective allosteric modulators. Without having carried out mutational studies – as for the other two orphan sites described in this work –, we can only hypothesise about the possible allosteric effect of a ligand that binds to this site. Here, our contact analysis could provide the reader with interesting insights into the possible receptor modulation through OS6.

From the analysis of the occurrence of crystallisation additives in the known sites, we were able to draw conclusions about the preferences of each. Overall, this analysis showed that fatty acids, in particular monoolein and oleic acid, are the most prevalent type of additives occupying the analysed known pockets and that KS5 is the pocket where they are located most frequently. This was to be expected, as the pockets are located in the membrane, but might on the other hand point towards a role of certain pockets in the regulation of function through lipid bilayer composition. A notable exception is KS11, which prefers anions.

Supplementary Figures

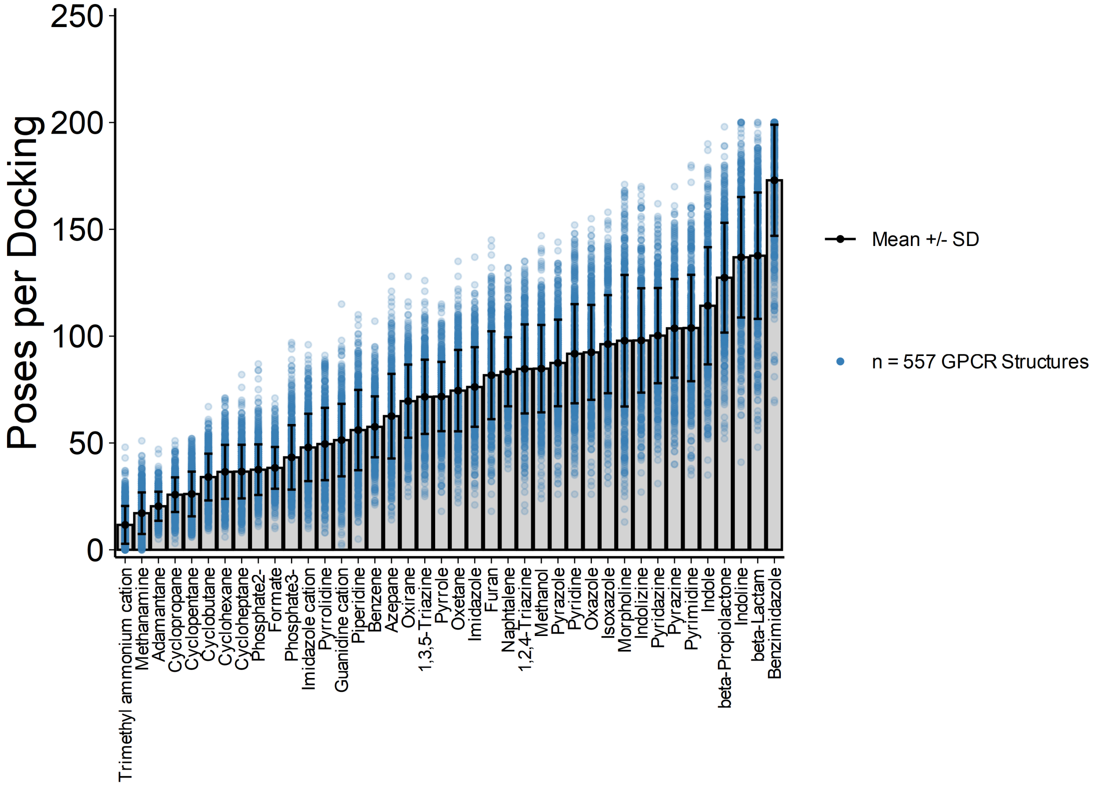

Supplementary Figure 1: Average number of poses per docking for each small molecular probe. Data is shown as mean values ± SD across n=557 docking calculations. Individual data points are shown as blue dots. Source data are provided as a source_data.xlsx file.

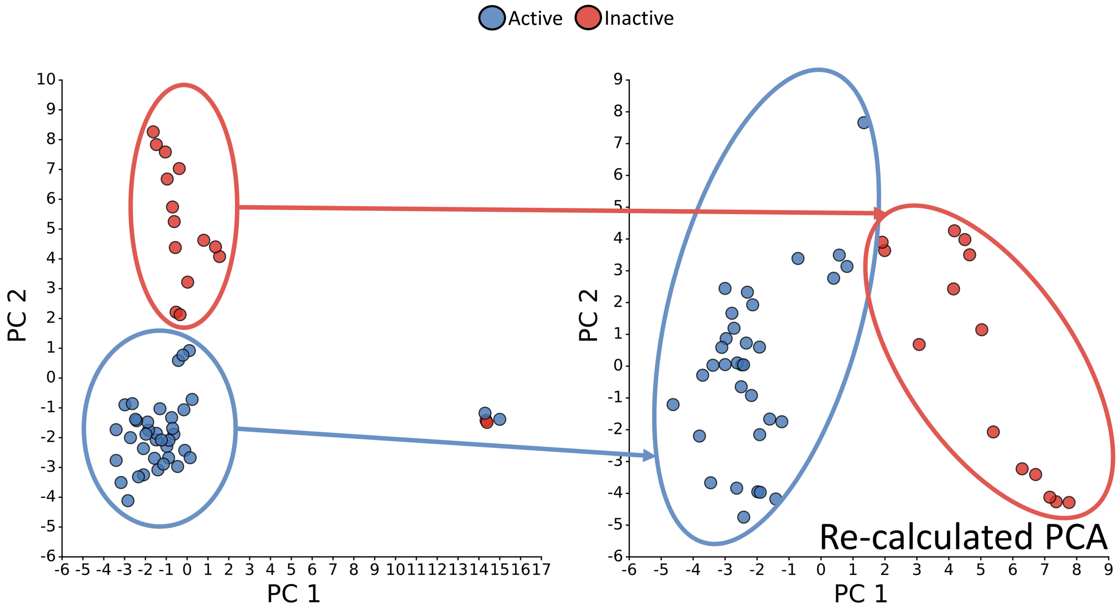

Supplementary Figure 2: Plot of the first two principal components (PCs) of the residue contact analysis for class B1 structures. Each point represents a PDB structure. They are colored according to the GPCRdb classification into active (blue), and inactive (red).[1] The right panel shows the data re-calculated based on the points in the clearly separated active and inactive clusters. Each PC value for each PDB can be seen as a linear combination of variables (i.e. contacts) that represents the residue contact landscape of a structure in a condensed manner. The first two PCs shown here explained most of the variance across all structural data, hence represent the most interesting PCs for investigating crucial differences between receptor states on the residue contact level. Source data are provided as a source_data.xlsx file.

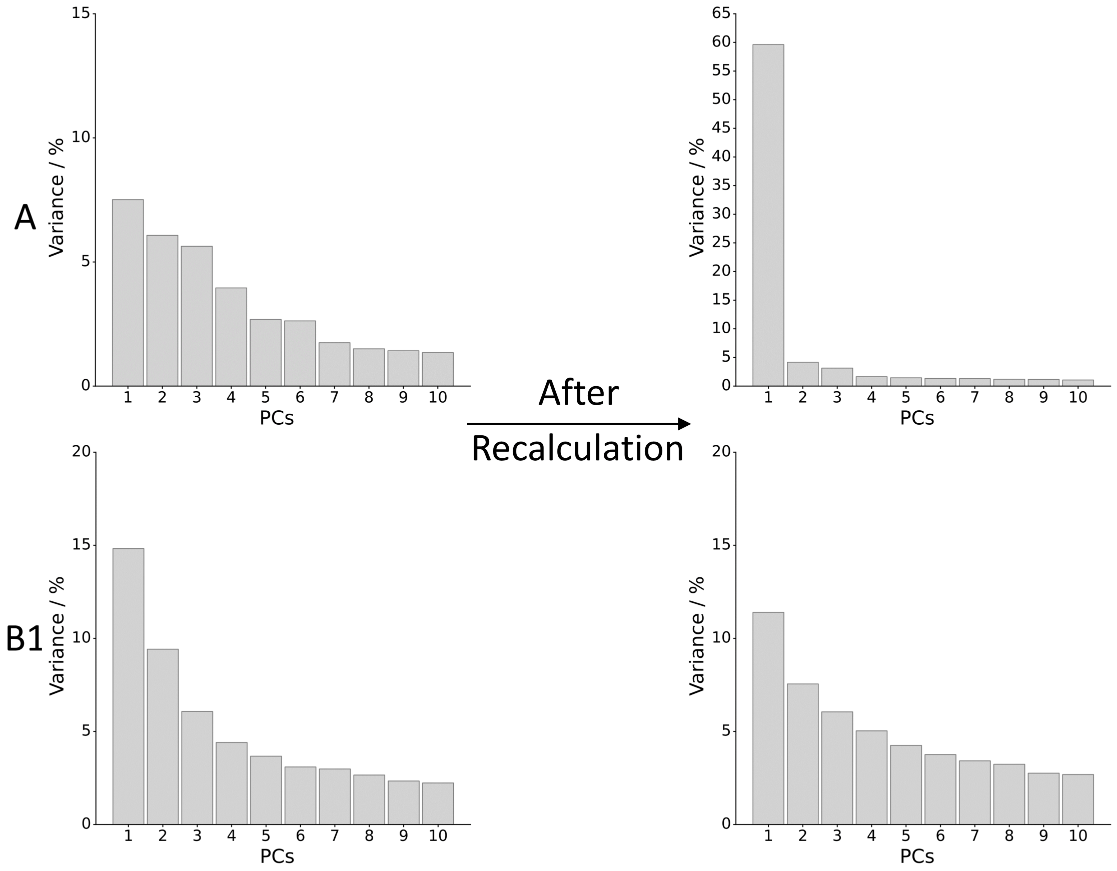

Supplementary Figure 3: Loadings of the first 10 principal components (PC) of the analysis depicted in Fig. 2 (class A GPCRs, upper panels) and Supplementary Figure 2 (class B1 GPCRs, lower panels). Right panels for each class correspond to the recalculated data based on the two best-defined clusters. Source data are provided as a source_data.xlsx file.

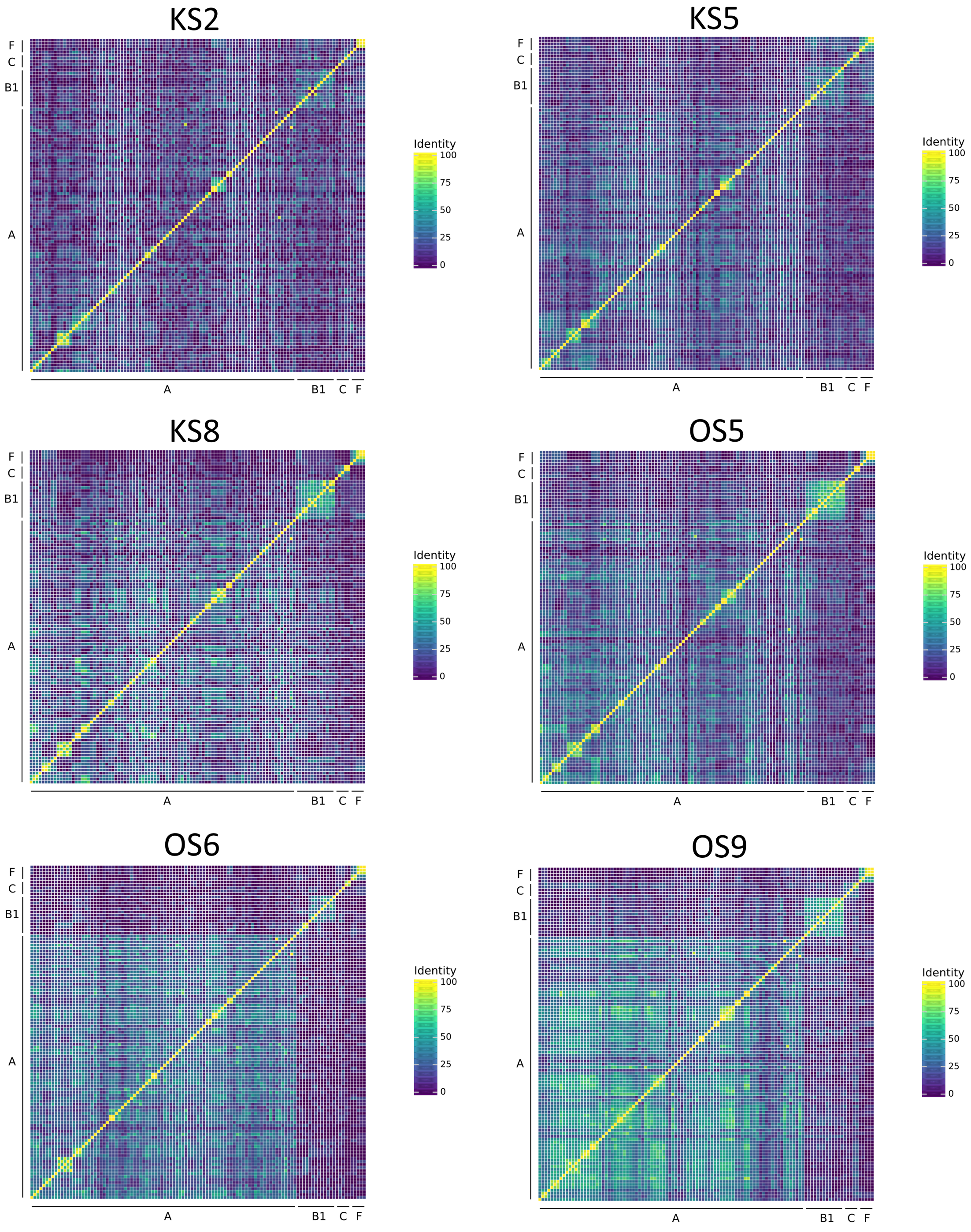

Supplementary Figure 4: Sequence identities for each of the named pockets across classes A, B1, C and F. Only sequences of structurally resolved and docked receptors were considered. Source data are provided as a source_data.xlsx file.

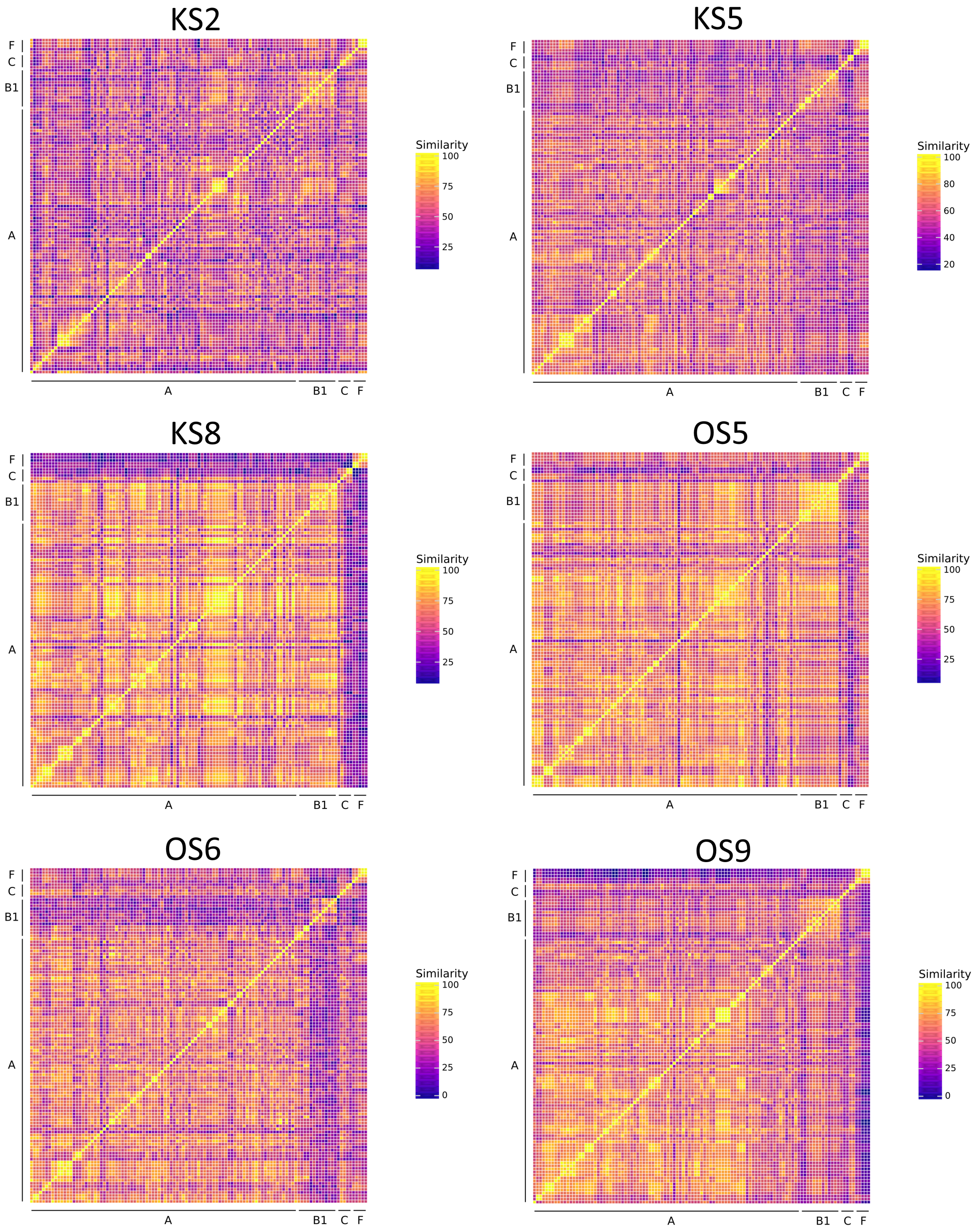

Supplementary Figure 5: Sequence similarities for each of the named pockets across classes A, B1, C and F. Only sequences of structurally resolved and docked receptors were considered. Source data are provided as a source_data.xlsx file.

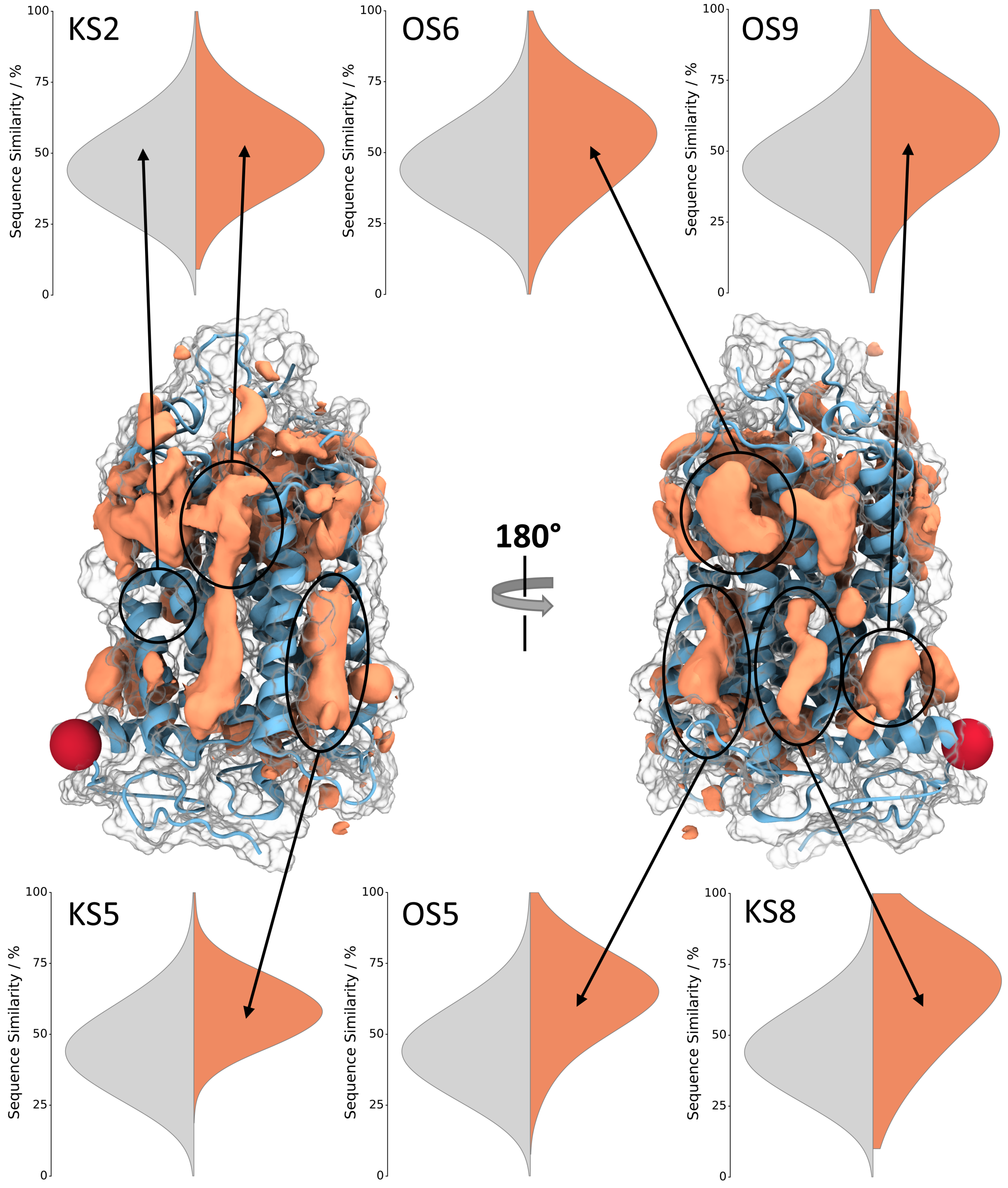

Supplementary Figure 6: The GPCR pocketome as shown in Fig. 1. For each of the three known (KS) and orphan sites (OS), the distribution of sequence similarities across the analysed receptorome (shown in Supplementary Figure 5) is depicted. Each of the distributions is directly compared against the sequence similarity distribution of a random stretch of residues across the receptorome (grey; this data is identical for each pocket). Source data are provided as a source_data.xlsx file.

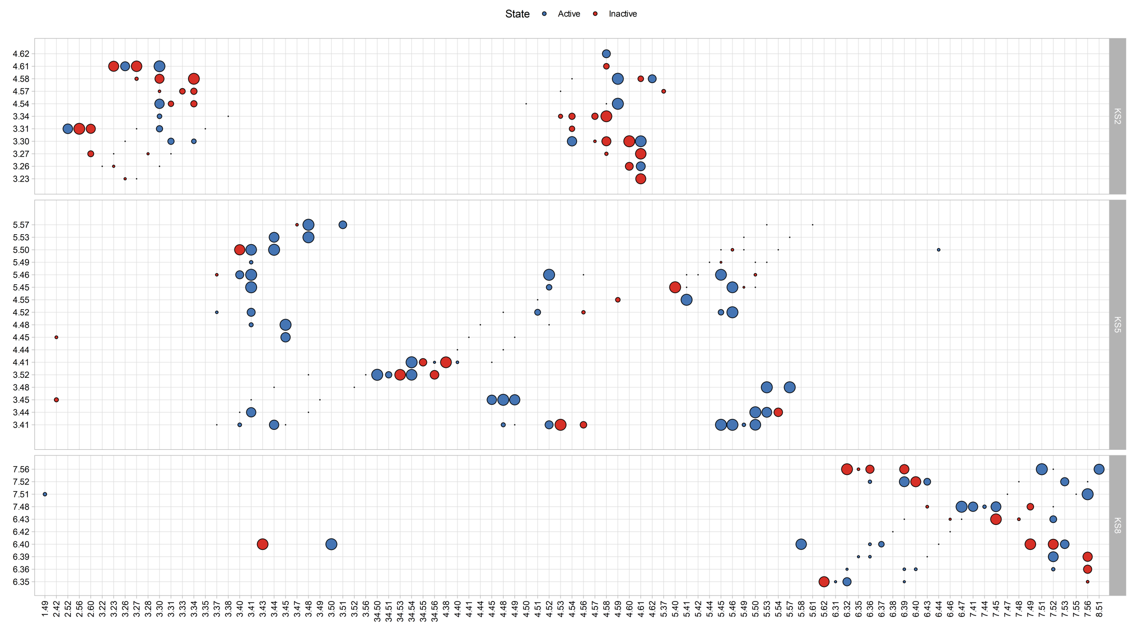

Supplementary Figure 7: State-specific residue contacts in three known sites of class A GPCR structures (Ballesteros-Weinstein numbering[2]). Points are colored according to the activation state (blue: active, red: inactive). The point size correlates with the normalised PCA coefficient for a given contact and can be seen as a direct measurement of importance for a given state. Source data are provided as a source_data.xlsx file.

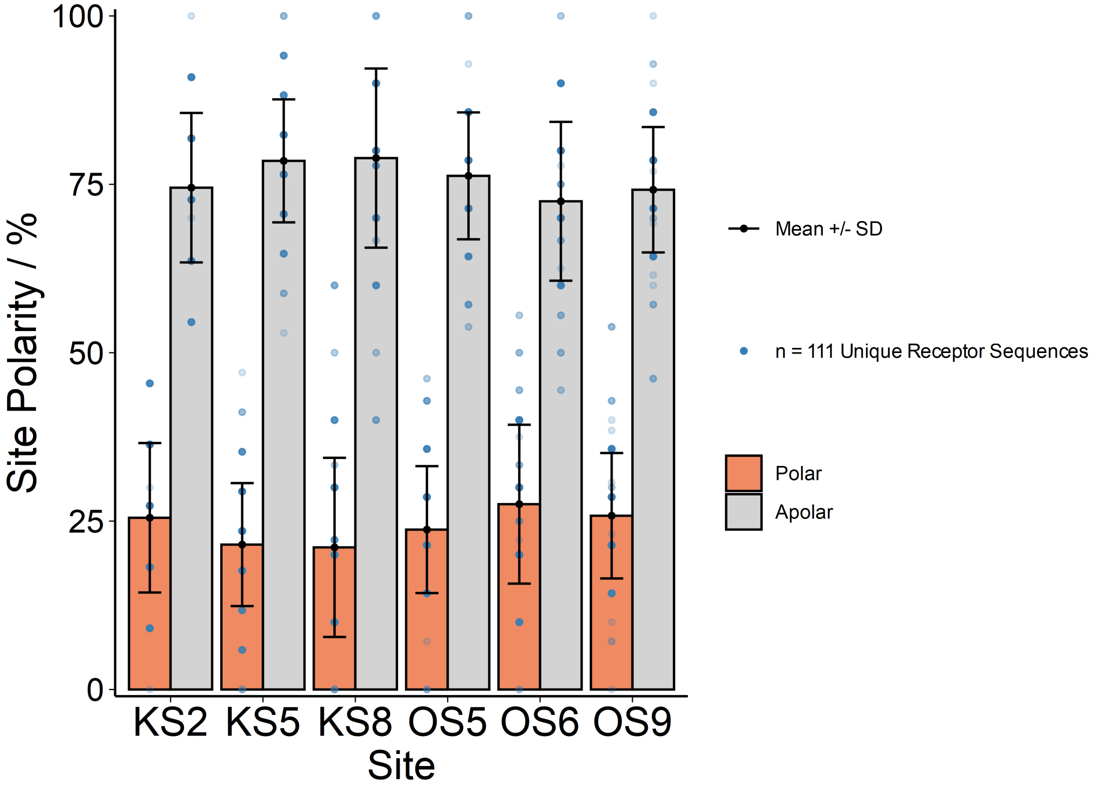

Supplementary Figure 8: Distribution of apolar and polar residues in each of the six sites discussed. Data is shown as mean values ± SD across n=111 unique receptor sequences. Individual data points are shown as blue dots. Source data are provided as a source_data.xlsx file.

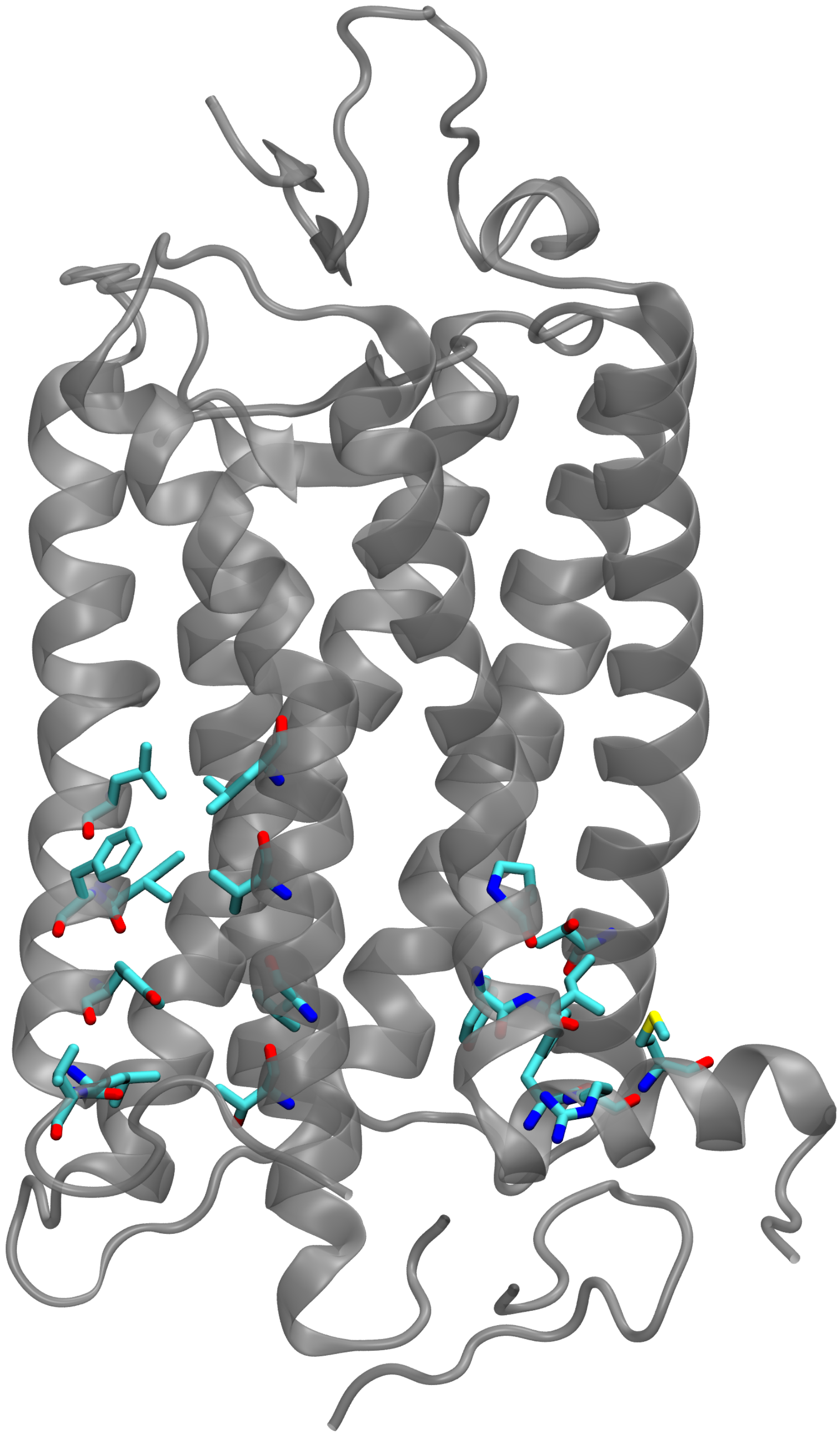

Supplementary Figure 9: Visualisation of the OS5 and OS9 residues mutated in the M_3R and β_2AR. The residues were selected based on their accessibility from the lipid bilayer. By carefully investigating different GPCR structures, we ensured that the majority of residues does not point their sidechain towards the G-protein binding site in order to exclude a direct interference with G-protein binding in the mutation experiments. Source data are provided as a source_data.xlsx file.

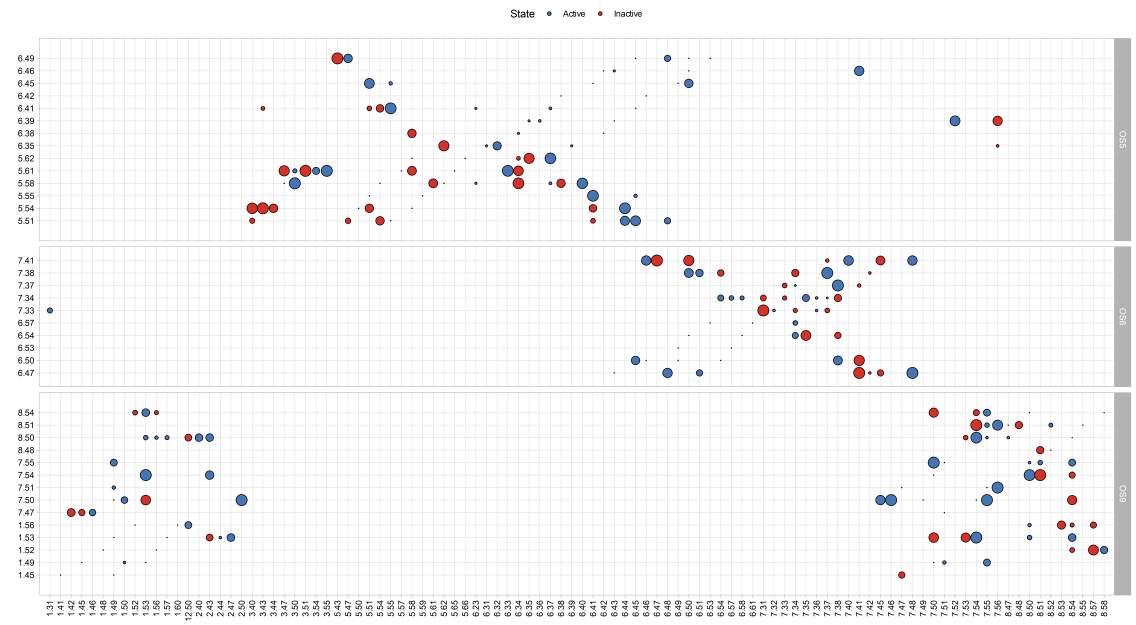

Supplementary Figure 10: State-specific residue contacts in three orphan sites of class A GPCR structures (Ballesteros-Weinstein numbering[2]). Points are colored according to the activation state (blue: active, red: inactive). The point size correlates with the normalised PCA coefficient for a given contact and can be seen as a direct measurement of importance for a given state. Source data are provided as a source_data.xlsx file.

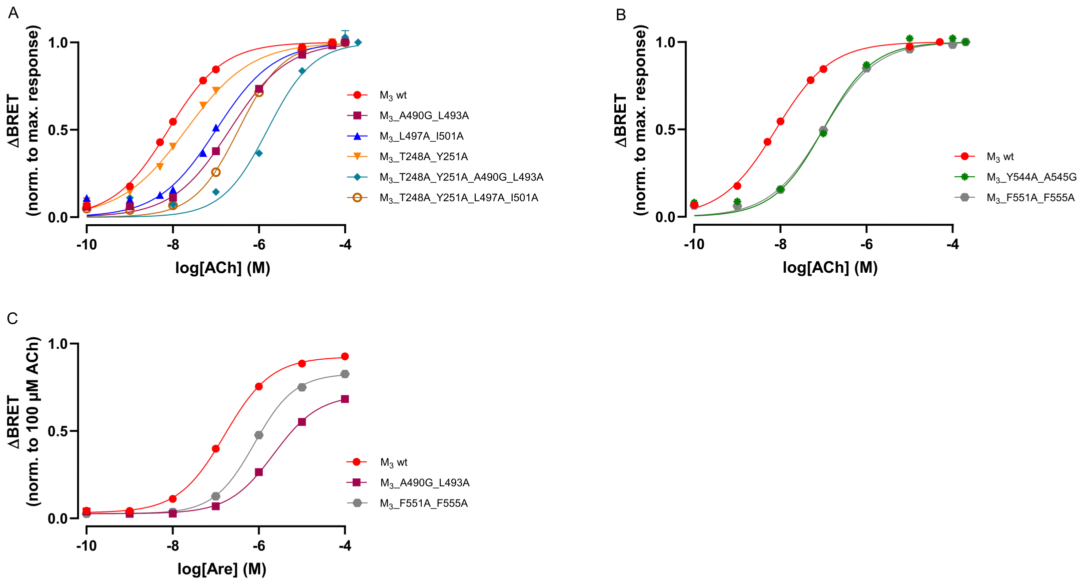

Supplementary Figure 11: M_3R-induced $G_{\alpha q}$ activation was measured by means of BRET between $G_{\alpha q}$-YFP and nLuc-G_{γ} in transiently transfected HEK293T cells. Every well was stimulated with a distinct concentration of agonist as indicated, afterwards with a saturating concentration of acetylcholine. The BRET ratio was normalised to the maximum response of acetylcholine of every well. Concentration-response curves of acetylcholine were plotted for M_3 receptor proteins with mutations in the region of (A) OS5 and (B) OS9 (data set for M_3R wt is identical in A and B n=10, M_3R_A490G_L493A n=12, M_3R_L497A_I501A n=12, M_3R_T248A_Y251A n=13, M_3R_T248A_Y251A_A490G_L493A n= 12, M_3R_T248A_Y251A_L497A_I501A n=12, M_3R_Y544A_A545G n=13, M_3R_F551A_F555A n=13 from at least 3 biologically independent experiments). (C) Concentration-response curves of arecoline were plotted for M_3R wt, OS5-mutated M_3R and OS9-mutated M_3R. (M_3R wt n= 15, M_3R_A490G_L493A n=15, M_3R_F551A_F555A n=15 from at least 3 biologically independent experiments). All data are shown as mean values ± SEM for each condition. Source data are provided as a source_data.xlsx file.

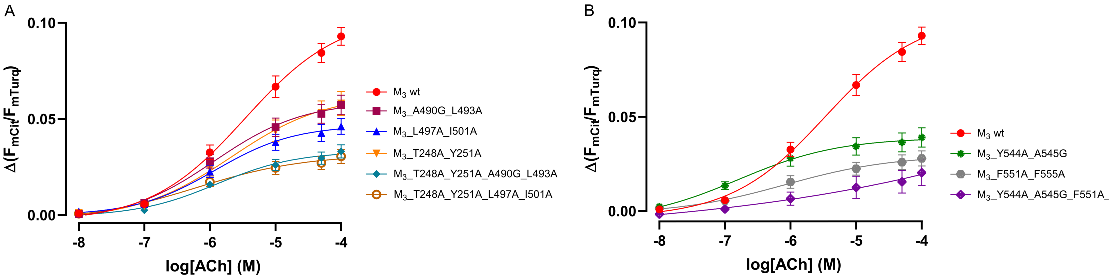

Supplementary Figure 12: Mutations of the M_3 receptors in regions of (A) OS5 and (B) OS9 affect β-arrestin2 recruitment. M_3R-mediated β-arrestin2 recruitment was measured in single cells by means of agonist-evoked FRET between mCitrine-tagged M_3 receptors and mTurquoise-tagged β-arrestin2. The cells were stimulated for 30 seconds with each concentration of acetylcholine. The changes in mCitrine/mTurquoise emission ratio of each concentration were plotted as mean values ± SEM for each condition. Data set shown for M_3R wt is identical in panels A and B. All measurements were corrected individually according to the determined expression ratio of fluorescent M_3 receptors and β-arrestin2 to account for the influence of the $^{M_3R}/_{\beta\text{-arrestin2}}$ expression ratio on the FRET amplitude (Supplementary Figure 16; M_3R wt n=14, M3_A490G_L493A n=11, M_3R_L497A_I501A n=8, M_3R_T248A_Y251A n=8, M_3R_T248A_Y251A_A490G_L493A n= 12, M_3R_T248A_Y251A_L497A_I501A n=11, M_3R_Y544A_A545G n=10, M_3R_F551A_F555A n=11, M_3R_Y544A_A545G_F551A_F555A n=9 biologically independent cells). Source data are provided as a source_data.xlsx file.

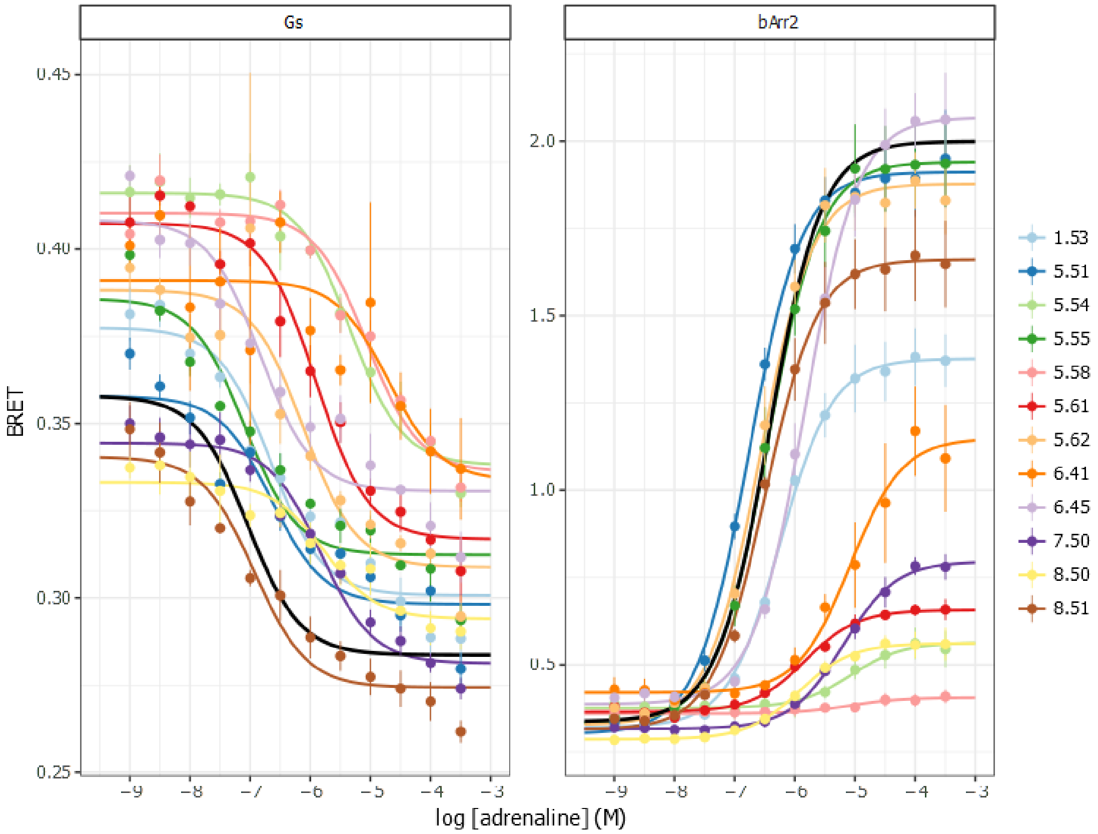

Supplementary Figure 13: Mutations of the β_2AR in regions of OS5 and OS9 affect $G_{\alpha s}$ activation and β-arrestin2 recruitment upon stimulation with adrenaline. Concentration-response curves for $G_{\alpha s}$ activation and β-arrestin2 recruitment are shown. All data are n=3, error bars are standard error of the mean (SEM). Source data are provided as a source_data.xlsx file.

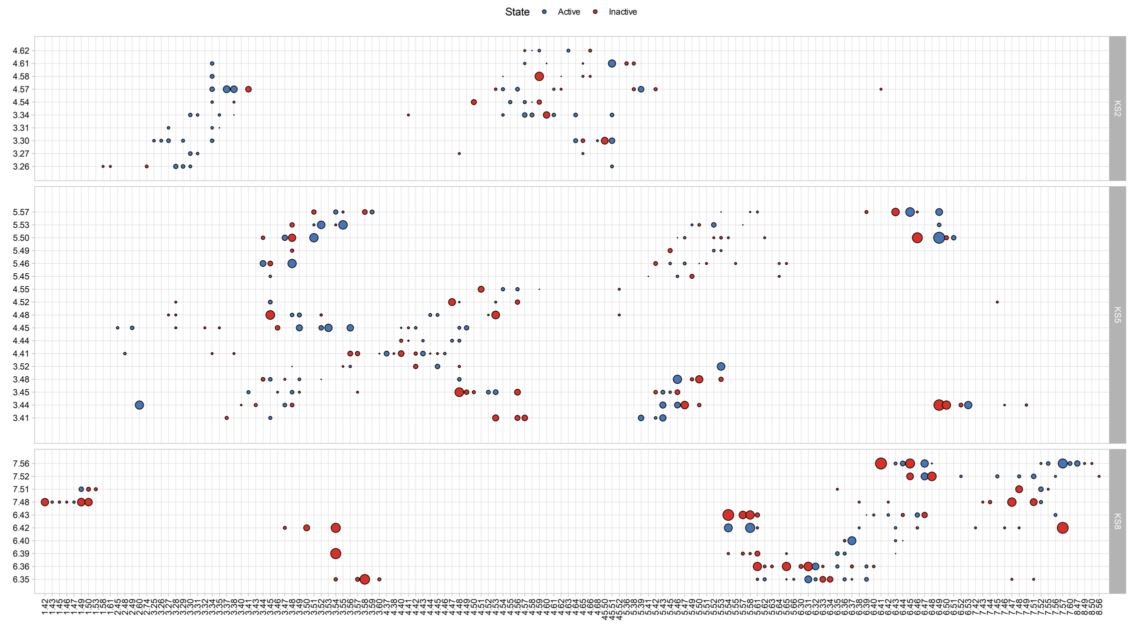

Supplementary Figure 14: State-specific residue contacts in three known sites of class B1 GPCR structures (Wootten numbering[3]). Points are colored according to the activation state (blue: active, red: inactive). The point size correlates with the normalised PCA coefficient for a given contact and can be seen as a direct measurement of importance for a given state. Source data are provided as a source_data.xlsx file.

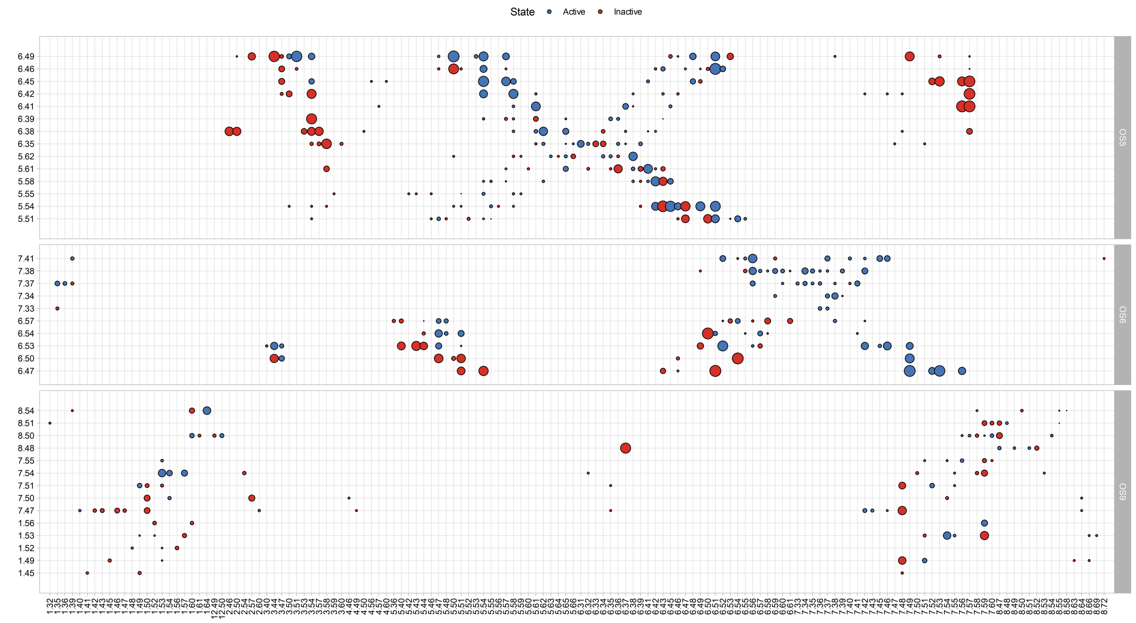

Supplementary Figure 15: State-specific residue contacts in three orphan sites of class B1 GPCR structures (Wootten numbering[3]). Points are colored according to the activation state (blue: active, red: inactive). The point size correlates with the normalised PCA coefficient for a given contact and can be seen as a direct measurement of importance for a given state. Source data are provided as a source_data.xlsx file.

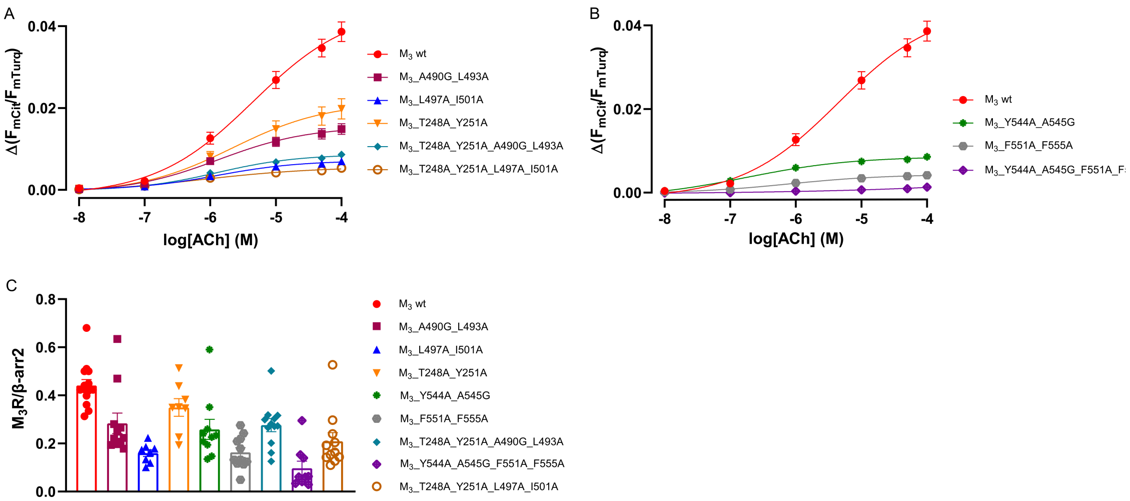

Supplementary Figure 16: FRET-based single-cell measurement of β-arrestin2 recruitment for OS5-mutated (A) and OS9-mutated (B) M_3 receptors. Shown are the uncorrected measurements of Supplementary Figure 12. (C) Different expression levels of the M_3 receptors in relation to β-arrestin2 were observed. The ratio of the mCitrine-tagged M_3R and the mTurquoise-tagged β-arrestin2 were determined for every cell and normalised to the mCitrine/mTurquoise-ratio of a reference construct (n=20) containing both fluorophores. The measurements of (A) and (B) were corrected for an equal expression ratio of M_3R and β-arrestin2 shown in Supplementary Figure 12. All data are shown as mean values ± SEM for each condition (M_3R wt n=14, M_3R_A490G_L493A n=11, M_3R_L497A_I501A n=8, M_3R_T248A_Y251A n=8, M_3R_T248A_Y251A_A490G_L493A n= 12, M_3R_T248A_Y251A_L497A_I501A n=11, M_3R_Y544A_A545G n=10, M_3R_F551A_F555A n=11, M_3R_Y544A_A545G_F551A_F555A n=9 biologically independent cells). Source data are provided as a source_data.xlsx file.

Supplementary Tables

Molecule	Name	SMILES
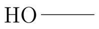	methanol	CO
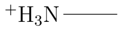	methanamine	C[NH3+]
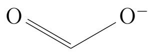	formate	C(=O)[O-]
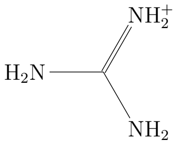	guanidine cation	C(=[NH2+])(N)N
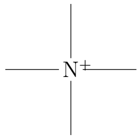	tetramethyl ammonium cation	C[N+](C)(C)C
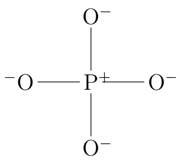	phosphate3-	[O-][P+]([O-])([O-])[O-]
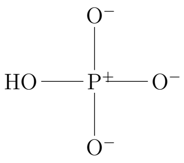	phosphate2-	[O-][P+]([O-])([O-])[OH]
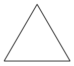	cyclopropane	C1CC1
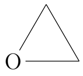	oxirane	C1CO1

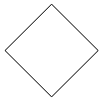	cyclobutane	C1CCC1
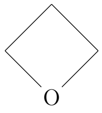	oxetane	C1COC1
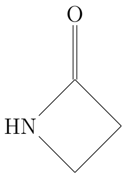	beta-lactam	C1(=O)CCN1
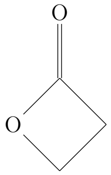	beta-propiolactone	C1(=O)CCO1
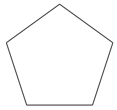	cyclopentane	C1CCCC1
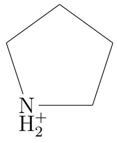	pyrrolidine	C1CC[NH2+]C1
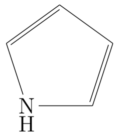	pyrrole	c1cc[nH]c1
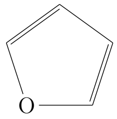	furan	c1ccoc1
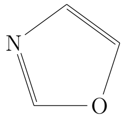	oxazole	c1cocn1

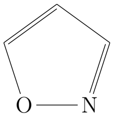	isoxazole	c1cnoc1
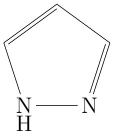	pyrazole	c1cn[nH]c1
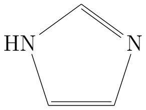	imidazole	c1c[nH]cn1
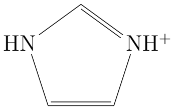	imidazole cation	c1c[nH+]c[nH]1
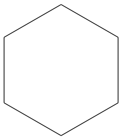	cyclohexane	C1CCCCC1
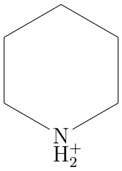	piperidine	C1CC[NH2+]CC1
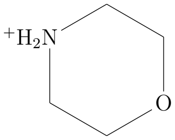	morpholine	C1COCC[NH2+]1
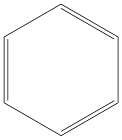	benzene	c1ccccc1
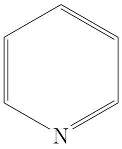	pyridine	c1ccncc1

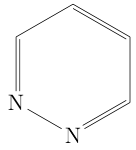	pyridazine	c1ccnnc1
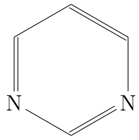	pyrimidine	c1cncnc1
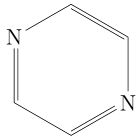	pyrazine	c1cnccn1
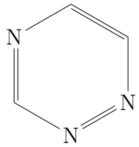	1,2,4-triazine	c1cnncn1
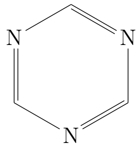	1,3,5-triazine	c1ncncn1
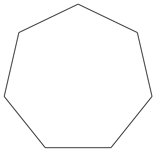	cycloheptane	C1CCCCCC1
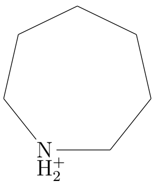	azepane	C1CCC[NH2+]CC1
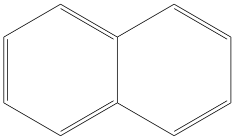	naphtalene	c1ccc2ccccc2c1

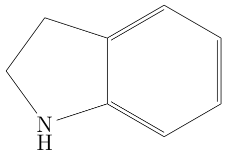	indoline	c1ccc2c(c1)CCN2
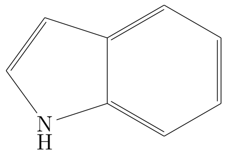	indole	c1ccc2c(c1)cc[nH]2
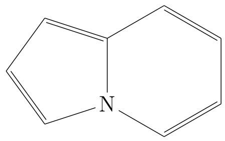	indolizine	c1cc2ccccn2c1
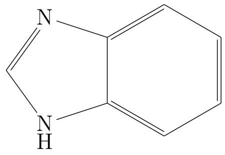	benzimidazole	c1ccc2c(c1)nc[nH]2
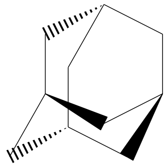	adamantane	C1[C@H]2C[C@H]3C[C@@H]1C[C@@H](C2)C3

Supplementary Table 1: Small molecular probes used for docking. Source data are provided as a source_data.xlsx file.

Site	Location[a]	Class	PDB ID[b]
OS1	UP OF I,II	A, B1, B2, C, D1, F	
OS2	LP OF I,II	A, B1, B2, C, D1	
OS4	MP OF V,VI	B1, B2, C, D1, F	
OS5	MP-LP OF V,VI	A, B1, B2, C, D1, F	
OS6	UP OF VI,VII	A, B1, C, F	
OS7	UP OF I,VII	A, B1, B2, C, D1, F	
OS8	MP OF I,VII	A, B1, B2, C, F	
OS9	LP OF I,VII,VIII	A, B1, B2, C, D1, F	
OS10	LP OF I,VIII	A, B1, C, F	
IBS1	UP-MP IF	A, B1, B2, C, D1, F	
IBS2	UP IF Above IBS1 IV,V, VI	A, B2, C	A (4MQT-ago, 4XNW-ant, 6RZ5-ant, 6RZ8-ant, 6RZ9-ant, 6U1N-ago)
IBS3	UP IF Next to IBS1 I,II,III,VII	A, B1, C, D1	A (3ODU-ant, 3OE6-ant, 3OE8-ant, 3OE9-ant, 3REY-ant, 5NDD-ant, 5T1A-ant, 5U09-invago, 5X33-ant, 5YHL-ant, 5YWY-ant, 6AK3-ago, 6GPS-ant, 6GPX-ant, 6IIU-ant, 6IIV-ant, 6LI0-ago, 6OIK-ago, 6RNK-ant, 6U1N-ago, 6Z10-ant), B1 (6ORV-ago, 6VCB-ago, 6X19-ago, 6X1A-ago, 6XOX-ago, 7C2E-ago)
SODIUM	MP IF I,II,III,VI,VII	A, B1, C, D1, F	A (5X33-ant), C (4OO9-NAM, 5CGC-NAM, 5CGD-NAM, 6FFH-NAM, 6FFI-NAM)
GPROT	LP IF II,III,V,VI	A, B1, B2, C, D1, F	
KS1	UP OF II,III	A, B1, B2, C, D1, F	A (4XNV-NAM)
KS2	UP OF III,IV	A, B1, B2, C, D1, F	A (4PHU-ago, 5NDZ-ant, 5TZR-ago, 5TZY-ago)
KS3	MP-LP OF II,IV	A, B1, B2, C, D1, F	A (6KQI-NAM)
KS4	UP OF IV,V	A, B1, B2, C, D1, F	A (6RZ5-ant, 6RZ8-ant, 6RZ9-ant)

KS5	MP-LP OF III,IV,V	A, B1, B2, C, D1, F	A (5KW2-ago, 5O9H-ant, 5TZY-PAM, 6C1Q-NAM, 6C1R-NAM, 6N48-PAM, 6OBA-NAM, 7CKZ-PAM, 7LJC-PAM, 7LJD-PAM)
KS6	LP OF IV,V	A, B1, C, D1, F	A (6C1R-NAM)
KS7	MP OF VI,VII	A, B1, B2, C, F	B1 (6KJV-NAM)
KS8	LP OF VI,VII	A, B1, C, D1, F	B1 (5EE7-NAM, 5VEW-NAM, 5VEX-NAM, 5XEZ-NAM, 5XF1-NAM, 6KJV-NAM, 6KK1-NAM, 6KK7-NAM, 6LN2-NAM), C (7CA3-PAM)
KS9	LP OF VII,VIII	A, B1, C, D1	A (5X7D-ant), B1 (5EE7-NAM, 5VEW-NAM, 5VEX-NAM, 5XEZ-NAM, 5XF1-NAM, 6KK1-NAM, 6KK7-NAM, 6LN2-NAM)
KS10	MP IF III,V,VI	B1, B2	B1 (4K5Y-ant, 4Z9G-ant)
KS11	LP IF I,II,VII,VIII	A, B1, B2, C, D1, F	A (5LWE-ant, 5T1A-ant, 5X7D-ant, 6LFL-ant, 6QZH-ant)
KS12	UP OF V,VI	A, B1, B2, C, D1, F	C (7M3J-NAM)

[a]LP: lower portion, UP: upper portion, MP: middle portion, IF: inward-facing, OF: outward-facing
[b]:-NAM: negative allosteric modulator, -PAM: positive allosteric modulator, -ago: agonist, -ant: antagonist, -invago: inverse agonist. Classification as provided by GPCRdb.[4]

Supplementary Table 2: List of all pockets observed after probe docking, their approximate locations and structures in which they are visible. Tags in italics after each PDB ID denote the effect of the allosteric ligand in the respective X-ray structure. We note that OS3 was described in the recent X-ray structure PDB 7M3J[5] [http://doi.org/10.2210/pdb7M3J/pdb] during writing of this manuscript. We therefore re-labeled this site to KS12. Source data are provided as a source_data.xlsx file.

Receptor group	Receptor ID	PDB ID	Pocket	Compounds ID
Opsins	Rhodopsin	2PED	KS7, KS8	HTG
Adrenoceptor	β_1AR	2VT4	KS10	SOG
Adrenoceptor	β_1AR	2Y01	KS5	Y01
Adrenoceptor	β_1AR	2Y02	KS5	Y01
Adrenoceptor	β_1AR	2Y03	KS5	Y01
Adrenoceptor	β_1AR	2Y04	KS5	Y01
Adrenoceptor	β_1AR	2YCX	KS2	SOG
Adrenoceptor	β_1AR	2YCY	KS2	SOG
Opsins	Rhodopsin	2Z73	KS7	BOG
Opsins	Rhodopsin	3AYM	KS11	BOG
Adrenoceptor	β_2AR	3D4S	KS5	OLC
Opsins	Rhodopsin	3OAX	KS7, KS8	HTG
Neurotensin	NTR1	4BUO	KS2	GLY
Opioid	NOP	4EA3	KS5	OLB
5-hydroxytryptamine	$5HT_{2B}$	4IB4	KS5	OLC, PEG
Frizzled	SMO	4JKV	KS1, KS3	OLC, OLA
Glucagon	GCGR	4L6R	KS2	PEG
Opioid	δOR	4N6H	KS5	OLA
5-hydroxytryptamine	$5HT_{2B}$	4NC3	KS1	OLC, PEG
P2Y	P2Y12	4NTJ	KS5	OLC
Metabotropic glutamate	mGlu5	4OO9	SODIUM	MES
Metabotropic glutamate	mGlu1	4OR2	KS1	CLR
Free fatty acid	FFA1	4PHU	KS2, KS5	OLC, 2YB
Opioid	δOR	4RWA	KS5	OLC
Opsin	Rhodopsin	4WW3	KS11	BOG
Neurotensin	NTR1	4XEE	KS4	1PE
P2Y	P2Y1R	4XNV	KS5	Y01
Metabotropic glutamate	mGlu5	5CGC	SODIUM	MES, 51D
Adrenoceptors	β_2AR	5D5A	KS2	BU1
Opioid	NOP	5DHG	KS2, KS2	OLA

Opioid	NOP	5DHH	KS2	OLA
Adrenoceptors	β_1AR	5F8U	KS2	CLR
Adenosine	$A_{2A}R$	5IU7	KS5	OLA
Adenosine	$A_{2A}R$	5IU8	KS11, KS5	HT0, OLA
Adenosine	$A_{2A}R$	5IUB	KS5	OLA
Adenosine	$A_{2A}R$	5K2A	KS5	OLA
Adenosine	$A_{2A}R$	5K2B	KS5	OLA
Adenosine	$A_{2A}R$	5K2C	KS5	OLA
Adenosine	$A_{2A}R$	5K2D	KS5	OLA
Chemokine	CCR9	5LWE	KS5	OLA
Adenosine	$A_{2A}R$	5NM4	KS1	OLA
Adenosine	$A_{2A}R$	5OLO	KS2	OLA
Adenosine	$A_{2A}R$	5OLV	KS5	OLA
Adenosine	$A_{2A}R$	5OLZ	KS5, KS1	OLA
Adenosine	$A_{2A}R$	5OM1	KS5	OLA
Adenosine	$A_{2A}R$	5OM4	KS5	OLA
5-hydroxytryptamine	$5HT_{2B}$	5TVN	KS11	PEG
Cannabinoid	CB1	5U09	KS5	PEG
Adenosine	$A_{2A}R$	5UIG	KS11	EDT
Adenosine	$A_{2A}R$	5VRA	KS5	OLA
Dopamine	D4	5WIV	KS5	OLA
Endothelin	ETb	5X93	KS2	OLC
Lysophospholipid	LPA	5XSZ	KS5	OLC
Cannabinoid	CB1	5TZY	KS2	CLR
5-Hydroxytryptamine	$5HT_{2A}$	6A93	KS2, KS3	CLR, 1PE
5-Hydroxytryptamine	$5HT_{2A}$	6A94	KS2	CLR
5-Hydroxytryptamine	$5HT_{2A}$	6BQH	KS2	CLR
Dopamine	D2	6CM4	KS10	OLA
Prostanoid	PGD2	6D27	SODIUM	PGE, PG0
Metabotropic glutamate	mGlu5	6FFH	KS2, SODIUM	OLC
Metabotropic glutamate	mGlu5	6FFI	KS5, SODIUM	OLC
Chemokine	CCR2A	6GPX	KS7, KS8	OLA

Adenosine	$A_{2A}R$	6GT3	KS5	OLA
Tachykinin	NK1R	6HLO	KS1, KS2, KS5, KS7, KS8	OLA, OLC
Tachykinin	NK1R	6HLP	KS5, KS7, KS8, KS1	OLA, OLC, PEG
Succinate	GPR91	6IBB	KS1, KS2	OLC
Endothelin	ETb	6IGK	KS2	OLC
Prostanoid	TP	6IIU	KS5	OLC
Adenosine	$A_{2A}R$	6JZH	KS1	OLA
Endothelin	ETb	6K1Q	KS2	OLC
Adrenoceptors	$\alpha_{2B}AR$	6K42	KS2	OLA
Cannabinoid	CB_1	6KQI	KS5	PEG
Adrenoceptors	$\alpha_{2C}AR$	6KUW	KS5	OLC
Chemokine	CXCR2	6LFM	KS2	CLR
Orphans	GPR52	6LI1	KS11	PEG
Endothelin	ETb	6LRY	KS7, KS8	CLR, PLM
Dopamine	D2	6LUQ	KS5	OLA
Melatonin	MT1	6ME2	KS7, KS8	OLA
Melatonin	MT1	6ME3	KS7, KS8	OLA
Melatonin	MT1	6ME5	KS7, KS8	OLA
Cannabinoid	CB_1	6N4B	KS5	CLR
Parathyroid hormone	PTH1R	6NBF	KS2, KS5	CLR, PLM
Parathyroid hormone	PTH1R	6NBH	KS2, KS5	CLR
Frizzeld	SMO	6O3C	KS5	OLB
Adrenoceptors	β_2AR	6OBA	KS2, KS5	CLR, M3J
Formylpeptide	FPR2	6OMM	KS7, KS8	PLM
Angiotensin	AT1R	6OS0	KS11	Cl
Angiotensin	AT1R	6OS1	KS5	OLC
Angiotensin	AT1R	6OS2	KS5, IBS3	OLC, NH2
Corticotropin-releasing factor	CRF1R	6PB0	KS2	CLR
Corticotropin-releasing factor	CRF1R	6PB1	KS7, KS8	CLR
Adrenoceptors	β_2AR	6PRZ	KS5	OLC
Adrenoceptors	β_2AR	6PS0	KS11, KS7, KS8	SO4
Adrenoceptors	β_2AR	6PS1	KS11	SO4

Adrenoceptors	β_2AR	6PS2	KS2, KS5	CLR, OLC, OLB
Adrenoceptors	β_2AR	6PS3	KS2, KS5	CLR, OLC
Adrenoceptors	β_2AR	6PS4	KS5	OLC
Adrenoceptors	β_2AR	6PS6	KS5	OLC
Adenosine	A_{2A}R	6PS7	KS5	OLA
Cannabinoid	CB_1	6PT0	IBS3	PLM
Succinate	SUCR1	6RNK	KS5	OLC
Leukotriene	CysLT2	6RZ4	KS5, KS7	OLC
Leukotriene	CysLT2	6RZ5	KS2, KS5, KS7	OLA
Leukotriene	CysLT2	6RZ6	KS1, KS5, KS7	OLC
Leukotriene	CysLT2	6RZ9	KS1, KS2, KS5, KS7	OLA, OLC
Adenosine	A_{2A}R	6S0Q	KS1, KS5	OLA
Orexin	OX1	6TO7	KS2, KS5	SOG
Orexin	OX1	6TOS	KS2	SOG
Orexin	OX1	6TOT	KS2	SOG, PG4
Orexin	OX1	6TP6	IBS3	NT5, PG4
Orexin	OX1	6TPG	KS5	OLA
Orexin	OX1	6TPJ	KS5, IBS3	PG4
Orexin	OX1	6TPN	KS2, KS5	OLA
Orexin	OX1	6TQ4	KS5, IBS3	SOG
Orexin	OX1	6TQ6	KS5, KS11	SOG, SO4
Orexin	OX1	6TQ7	KS7, IBS3	SOG, NVK
Orexin	OX1	6TQ9	KS7, IBS3	SOG, NVK
Calcitonin	AM	6UUN	KS2	CLR
Orexin	OX1	6V9S	KS1, IBS3	CLR, JHC
Melanocortin	MC4R	6W25	KS7, KS8	OLA
Succinate	GPR91	6Z10	KS5	OLC
Adenosine	A_{2A}R	6ZDR	KS5	OLA
Bile acid	GPBA	7CFM	KS5	CLR
Dopamine	D1	7JVP	KS2	CLR
Dopamine	D1	7LJC	KS2	CLR

Supplementary Table 3: List of GPCR structures with a ligand different than a possible synthetic allosteric ligand in the corresponding binding pocket. Source data are provided as a source_data.xlsx file.

**Substructure**	**SMARTS**
Hydrogen bond donors	[$([N;!H0;v3,v4&+1]),$([O,S;H1;+0]),n&H1&+0]
Hydrogen bond acceptors	[$([O,S;H1;v2;!$(*-*=[O,N,P,S])]),$([O,S;H0;v2]),$([O,S;-]),$([N;v3;!$(N-*=[O,N,P,S])]),n&H0&+0,$([o,s;+0;!$([o,s]:n);!$([o,s]:c:n)])]
Aromatic atoms	[a]
Halogens	[F,Cl,Br,I]
Basic substructures	[#7;+,$([N;H2&+0][$([C,a]);!$([C,a](=O))]),$([N;H1&+0]([$([C,a]);!$([C,a](=O))])[$([C,a]);!$([C,a](=O))]),$([N;H0&+0]([C;!$(C(=O))])([C;!$(C(=O))])[C;!$(C(=O))])]
Acidic substructures	[$([C,S](=[O,S,P])-[O;H1,-1])]
Aliphatic rings	[R!a]
Everything	[*]

Supplementary Table 4: Substructures used for extraction of molecular features. Source data are provided as a source_data.xlsx file.

5’ CAAGGAGAAGAAAGCGGGCCAGACCGCCAGTGCGATCTTGCTTG 3’	(M3_A490G_L493A)
5’ ACCCTCAGTGCGATCGCGCTTGCCTTCGCCATCACTTGGACCCC 3’	(M3_L497A_I501A)
5’ GCCTGTCACCATTATGGCTATTTTAGCCTGGAGGATCTATAAGG 3’	(M3_T248A_Y251A)
5’ GTGAACCCCGTGTGCGCTGGTCTGTGCAACAAAACA 3’	(M3_Y544A_A545G)
5’ CTGTGCAACAAAACAGCCAGAACCACTGCCAAGATGCTGCTGCTG 3’	(M3_F551A_F555A)
5’ CGTGCTGGCCATCACCGCCATTGC 3’	(B2_V54A_f)
5’ GCGGTGATGGCCAGCACGTTGCCGAAC 3’	(B2_V54A_r)
5’ CGTGCCCGCCGTGATCATGGTGTTC 3’	(B2_L212A_f)
5’ ATGATCACGGCGGGCACGTAGAAGGAC 3’	(B2_L212A_r)
5’ TCGCCGTGTTCGTGTACAGCCGGGTG 3’	(B2_M215A_f)
5’ TGTACACGAACACGGCGATCACGAGGGGCAC 3’	(B2_M215A_r)
5’ TCATGGCCTTCGTGTACAGCCGGGTG 3’	(B2_V216A_f)
5’ TGTACACGAAGGCCATGATCACGAGGGGCAC 3’	(B2_V216A_r)
5’ CGTGGCCAGCCGGGTGTTCCAAG 3’	(B2_Y219A_f)
5’ AACACCCGGCTGGCCACGAACACCATGATC 3’	(B2_Y219A_r)
5’ CGTGTACGCCCGGGTGTTCCAAGAAG 3’	(B2_S220A_f)
5’ AACACCCGGGCGTACACGAACACCATG 3’	(B2_S220A_r)
5’ GGTGGCCCAAGAAGCCAAGCGGCAG 3’	(B2_F223A_f)
5’ TTGGCTTCTTGGGCCACCCGGCTGTACAC 3’	(B2_F223A_r)
5’ GGCATCATCGCCGGCACCTTCACTCTG 3’	(B2_M279A_f)
5’ GGTGCCGGCGATGATGCCCAGGGTTTTC 3’	(B2_M279A_r)
5’ CTTCGCTCTGTGCTGGCTGCCCTTC 3’	(B2_T283A_f)
5’ AGCCAGCACAGAGCGAAGGTGCCCATGATG 3’	(B2_T283A_r)
5’ CGGCTTCAACGCTCTGATCTACTGCAG 3’	(B2_P323A_f)
5’ ATCAGAGCGTTGAAGCCGCTGTTCACG 3’	(B2_P323A_r)
5’ CCCGACGCCCGGATCGCCTTCCAAG 3’	(B2_F332A_f)
5’ GGCGATCCGGGCGTCGGGGCTTCTGCAG 3’	(B2_F332A_r)
5’ CCCGACTTCGCCATCGCCTTCCAAGAG 3’	(B2_R333A_f)
5’ GGCGATGGCGAAGTCGGGGCTTCTG 3’	(B2_R333A_r)

Supplementary Table 5: Primers used to introduce the mutations into the M_3R and β_2AR, respectively. The substrings “_f” and “_r” denote forward and reverse primers, respectively.

References

[1] Kooistra, A. J. *et al.* GPCRdb in 2021: integrating GPCR sequence, structure and function. *Nucleic Acids Res.* **49**, D335–D343 (2020).

[2] Ballesteros, J. A. & Weinstein, H. Integrated methods for the construction of three-dimensional models and computational probing of structure-function relations in G protein-coupled receptors. In Sealfon, S. C. (ed.) *Receptor Molecular Biology*, vol. 25 of *Methods in Neurosciences*, chap. 19, 366–428 (Academic Press, San Diego, CA, 1995).

[3] Wootten, D., Simms, J., Miller, L. J., Christopoulos, A. & Sexton, P. M. Polar transmembrane interactions drive formation of ligand-specific and signal pathway-biased family B G protein-coupled receptor conformations. *Proc. Natl. Acad. Sci. U. S. A.* **110**, 5211–5216 (2013).

[4] Munk, C. *et al.* GPCRdb: the G protein-coupled receptor database–an introduction. *Br. J. Pharmacol.* **173**, 2195–2207 (2016).

[5] Gao, Y. *et al.* Asymmetric activation of the calcium-sensing receptor homodimer. *Nature* **595**, 455–459 (2021).
