# Supplementary material for: The pocketome of G-protein-coupled receptors reveals previously untargeted allosteric sites
Source: Nat Commun. 2022 May 10;13:2567. doi: 10.1038/s41467-022-29609-6 (PMC9091257; doi:10.1038/s41467-022-29609-6)
Supplement: Supplementary file 3 — Description of Additional Supplementary Files [file 41467_2022_29609_MOESM3_ESM.pdf]

Description of Additional Supplementary Files

File name: Supplementary Data 1

Description: list of all analysed structures together with the docking files

File name: Supplementary Data 2

Description: grid files, template and README for visualising the class-specific density maps by using Pymol
