# Supplementary material for: The pocketome of G-protein-coupled receptors reveals previously untargeted allosteric sites
Source: Nat Commun. 2022 May 10;13:2567. doi: 10.1038/s41467-022-29609-6 (PMC9091257; doi:10.1038/s41467-022-29609-6)

README

Introductory Notes

To properly display the class-specific density maps (”.dx” files), we advise the reader to use PyMOL. The instructions in this README were developed with Open-Source PyMOL Version 2.3.0. Please note that the GUI and function/command names might differ for different versions of PyMOL. The next Figure shows the start screen of a properly working version of PyMOL.

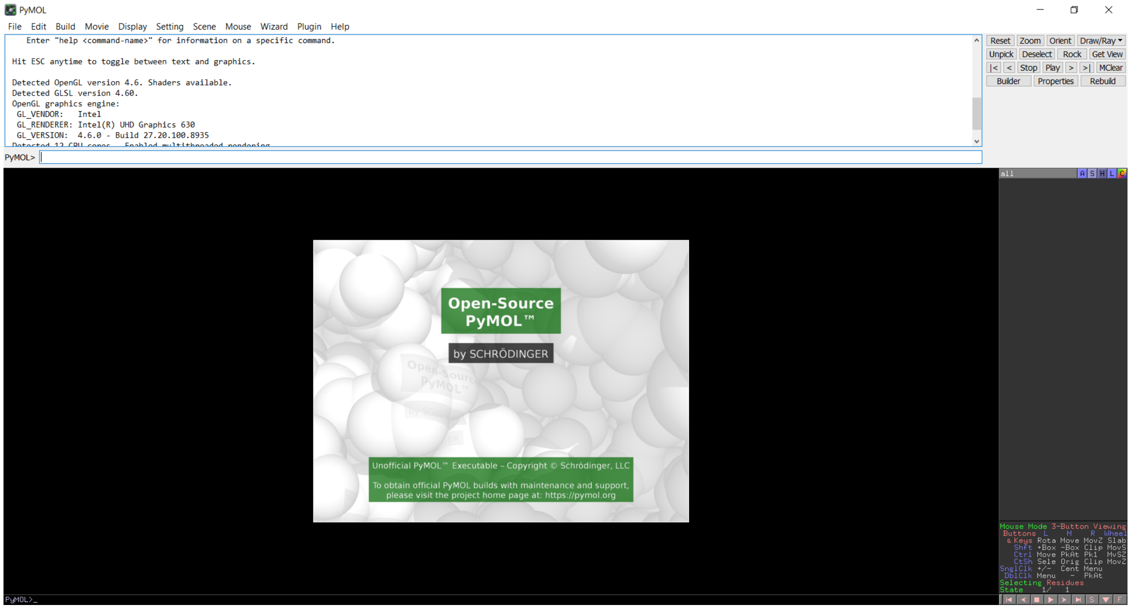

Loading the Files

The files provided are the complete class-specific density maps as discussed in this work. For orientation, a template structure is provided as a ”.mol2” file (template.mol2, PDB: 1F88). If the reader wishes to align their own receptor structures to these maps, they can do this by loading their structure and align it to the provided template structure. Via ”File → Open...” the reader can load the ”.dx” files and the ”.mol2” file directly into PyMOL.

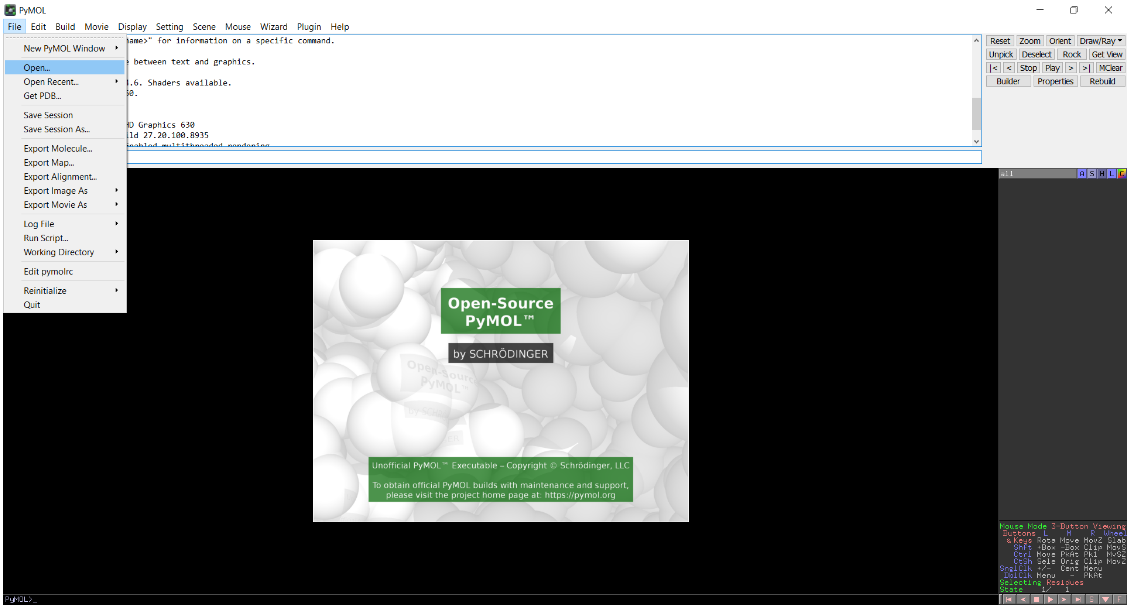

After loading the files, they should appear as objects on the right of the PyMOL GUI window. Furthermore, the template receptor structure should be visible. If this is not the case, please activate the structure by clicking on the object name on the right of the window.

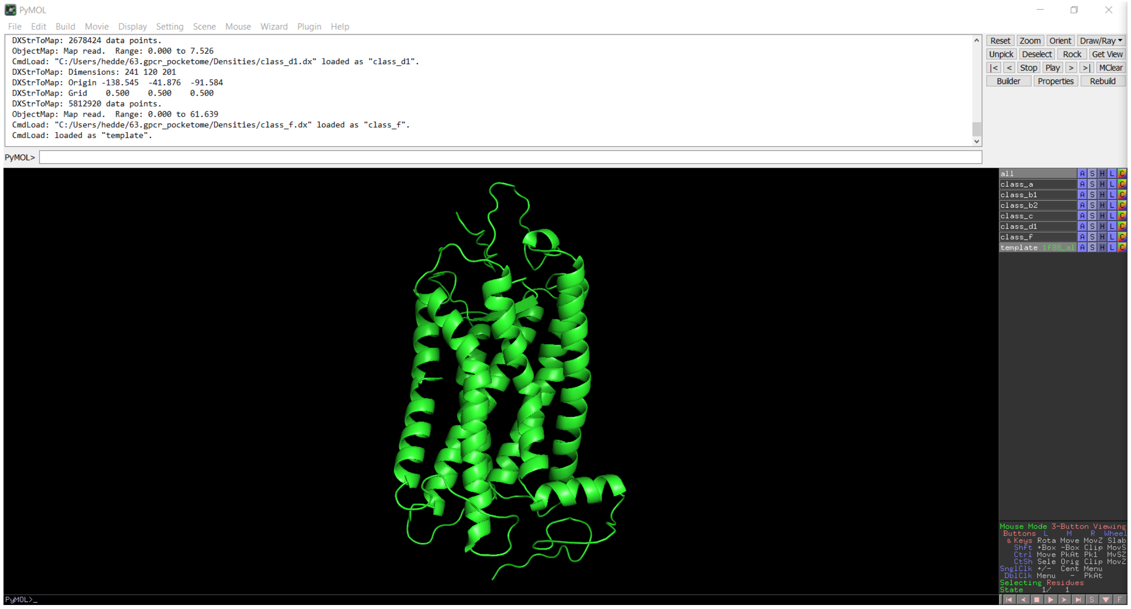

Showing the Density Maps

Next, show the density map of choice (e.g. the class A density map) by typing into the command line:

volume class_a_volume, class_a

This will create a new volume object called "volume_class_a" which then appears as an object to the right of the window. If it does not show up, please investigate whether the command was typed in correctly. As before, please also check whether the object is activated or not.

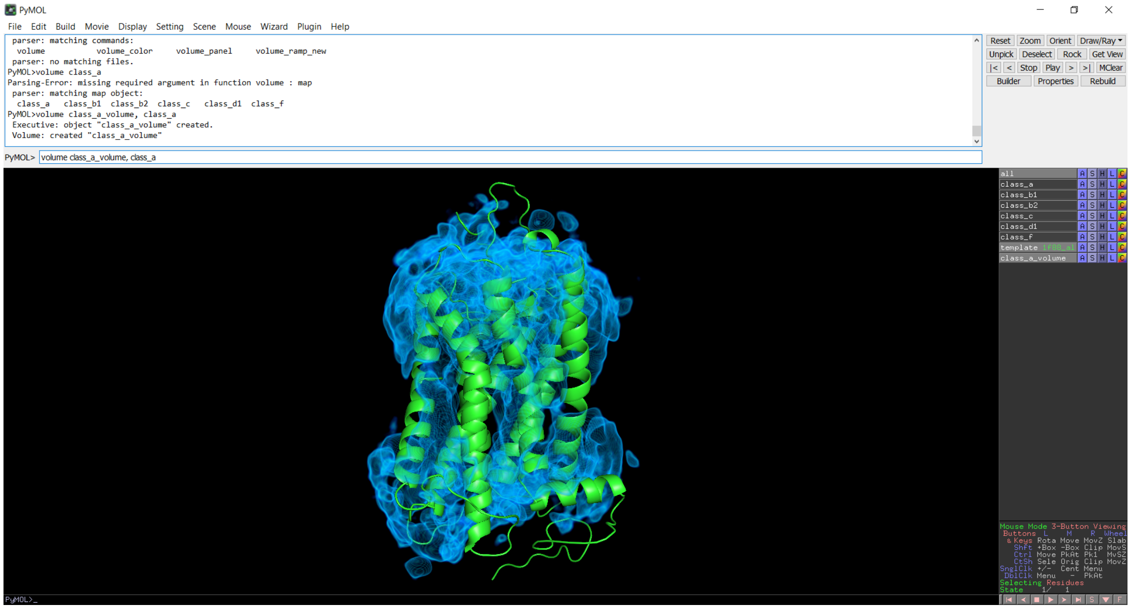

Finally, adjust the densities by opening the volume colour panel. You will find this next to the volume object "class_a_volume" as a rainbow-coloured box with a "C" inside. Click on it and chose "panel".

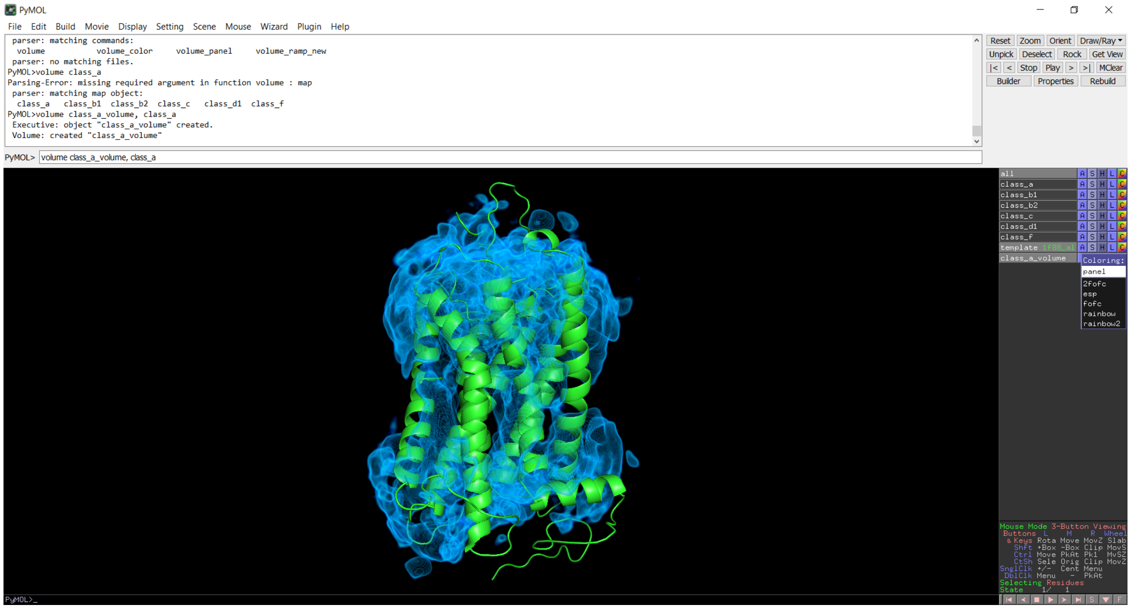

Then, adjust the x-axis cut-off, y-axis alpha value and colours according to your preferences. The points can be dragged around by using the left mouse button. Furthermore,

points can be added or removed also by using the left mouse button or by clicking the mouse wheel, respectively. We advise the user to use an x-axis cut-off of approximately 80% of the maximum value (here: 35-44). This will ensure that the hotspots will be clearly visible.

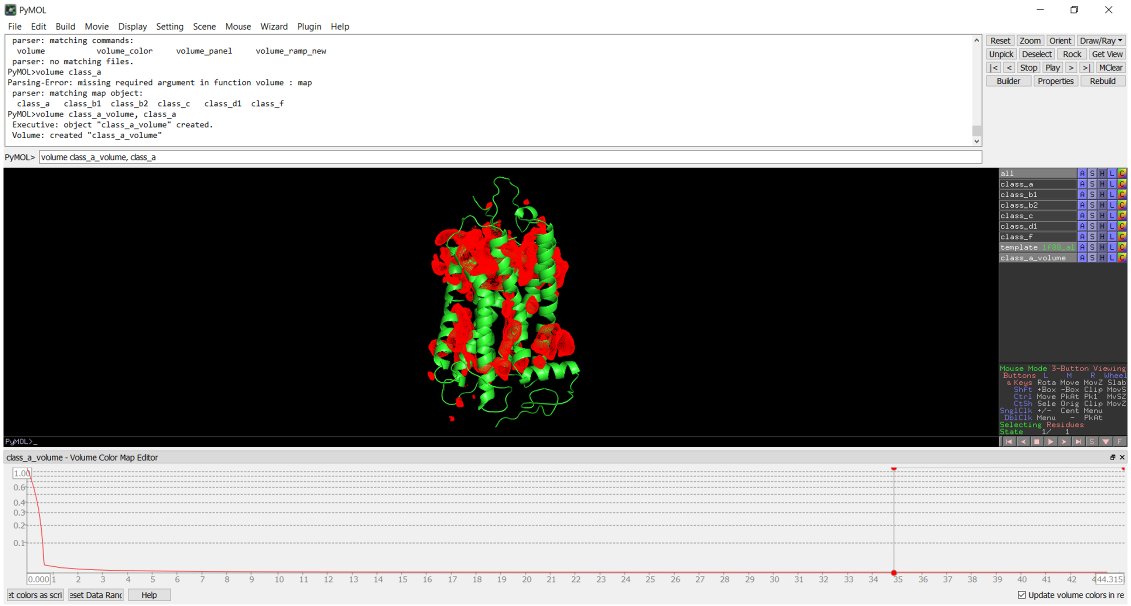

Supplement: Supplementary file 5 — Supplementary Data 2 [file 41467_2022_29609_MOESM5_ESM.zip › README.pdf]
