# Supplementary material for: The pocketome of G-protein-coupled receptors reveals previously untargeted allosteric sites
Source: Nat Commun. 2022 May 10;13:2567. doi: 10.1038/s41467-022-29609-6 (PMC9091257; doi:10.1038/s41467-022-29609-6)
Supplement: Supplementary file 6 — Reporting Summary [file 41467_2022_29609_MOESM6_ESM.pdf]

nature portfolio

Corresponding author(s): Peter Kolb

Last updated by author(s): Feb 21, 2022

Reporting Summary

Nature Portfolio wishes to improve the reproducibility of the work that we publish. This form provides structure for consistency and transparency in reporting. For further information on Nature Portfolio policies, see our Editorial Policies and the Editorial Policy Checklist.

Statistics

For all statistical analyses, confirm that the following items are present in the figure legend, table legend, main text, or Methods section.

n/a	Confirmed	
☐	☒	The exact sample size (*n*) for each experimental group/condition, given as a discrete number and unit of measurement
☐	☒	A statement on whether measurements were taken from distinct samples or whether the same sample was measured repeatedly
☒	☐	The statistical test(s) used AND whether they are one- or two-sided<br>*Only common tests should be described solely by name; describe more complex techniques in the Methods section.*
☒	☐	A description of all covariates tested
☒	☐	A description of any assumptions or corrections, such as tests of normality and adjustment for multiple comparisons
☐	☒	A full description of the statistical parameters including central tendency (e.g. means) or other basic estimates (e.g. regression coefficient) AND variation (e.g. standard deviation) or associated estimates of uncertainty (e.g. confidence intervals)
☒	☐	For null hypothesis testing, the test statistic (e.g. *F*, *t*, *r*) with confidence intervals, effect sizes, degrees of freedom and *P* value noted<br>*Give P values as exact values whenever suitable.*
☒	☐	For Bayesian analysis, information on the choice of priors and Markov chain Monte Carlo settings
☒	☐	For hierarchical and complex designs, identification of the appropriate level for tests and full reporting of outcomes
☒	☐	Estimates of effect sizes (e.g. Cohen's *d*, Pearson's *r*), indicating how they were calculated

Our web collection on statistics for biologists contains articles on many of the points above.

Software and code

Policy information about availability of computer code

Data collection	AAScan for primer design: https://github.com/dmitryveprintsev/AAScan; SparkControl; NIS-Elements AR; python (3.7) scripts based on the packages requests (2.25) and urllib3 (1.25.11)
Data analysis	RStudio (1.4.1717 [chemoinformatics] & 2021.09.2+382 [b2AR data]). R (4.1.2) and R packages tidyverse (1.3.1), drc (3.0-1). Graphpad Prism 8.3, Microsoft Excel 2019, OriginPro 2018, BRET2DTF, DataFitter, BMPViewer (the last three are custom in-house software, which we will of course make available). Scripts based on python packages pandas (1.1.4), biopython (1.78), biopandas (0.2.7) and numpy (1.19.4). RDkit (2020.09.1.0). VMD (1.9.3). open-source PyMol (2.3.0). Scikit-learn (0.23.2). Custom volumetric averaging code https://github.com/torbengutermuth/volumetricaveraging

For manuscripts utilizing custom algorithms or software that are central to the research but not yet described in published literature, software must be made available to editors and reviewers. We strongly encourage code deposition in a community repository (e.g. GitHub). See the Nature Portfolio guidelines for submitting code & software for further information.

Data

Policy information about availability of data

All manuscripts must include a data availability statement. This statement should provide the following information, where applicable:

- Accession codes, unique identifiers, or web links for publicly available datasets
- A description of any restrictions on data availability
- For clinical datasets or third party data, please ensure that the statement adheres to our policy

The list of the 557 structures, aligned receptor coordinate files, and the probe docking data generated in this study are provided as Supplementary Data 1. Separate pymol sessions of the pocket densities for each class are provided as Supplementary Data 2. Instead of being directly provided with this paper, they have been

deposited in the Zenodo database under accession code https://doi.org/10.5281/zenodo.5973911. The experimental receptor activation data generated in this study are provided in the Supplementary Information and as Source Data in the source data.xlsx file.

Field-specific reporting

Please select the one below that is the best fit for your research. If you are not sure, read the appropriate sections before making your selection.

☒ Life sciences ☐ Behavioural & social sciences ☐ Ecological, evolutionary & environmental sciences

For a reference copy of the document with all sections, see nature.com/documents/nr-reporting-summary-flat.pdf

Life sciences study design

All studies must disclose on these points even when the disclosure is negative.

Sample size	Sample sizes are the numbers n of repeat experiments. For b2AR, n=3; for M3R n varies, up to n=15. No samples size calculation was performed.
Data exclusions	No data were excluded.
Replication	All points measured in the assays were repeated multiple times and the mean +/- s.e.m. calculated. Samples are biological triplicates (measured 3 separate days using separately transfected cells). All replications were successful.
Randomization	All assays were performed in HEK cell lines, randomization was not necessary
Blinding	All assays were performed in HEK cell lines, blinding was not necessary

Reporting for specific materials, systems and methods

We require information from authors about some types of materials, experimental systems and methods used in many studies. Here, indicate whether each material, system or method listed is relevant to your study. If you are not sure if a list item applies to your research, read the appropriate section before selecting a response.

Materials & experimental systems

n/a	Involved in the study	
☒	☐	Antibodies
☐	☒	Eukaryotic cell lines
☒	☐	Palaeontology and archaeology
☒	☐	Animals and other organisms
☒	☐	Human research participants
☒	☐	Clinical data
☒	☐	Dual use research of concern

Methods

n/a	Involved in the study	
☒	☐	ChIP-seq
☒	☐	Flow cytometry
☒	☐	MRI-based neuroimaging

Eukaryotic cell lines

Policy information about cell lines

Cell line source(s)	HEK293T: a kind gift from the Lohse laboratory, University of Würzburg, originally ATCC CRL-3216; (HEK)-293 SL: a kind gift from Stephan Laporte, originally from ATCC
Authentication	None of the cell lines were authenticated
Mycoplasma contamination	Tested for the absence of contamination once per month.
Commonly misidentified lines (See ICLAC register)	None were used.
